# Global gene expression in cotton (*Gossypium hirsutum* L.) leaves to waterlogging stress

**DOI:** 10.1371/journal.pone.0185075

**Published:** 2017-09-27

**Authors:** Yanjun Zhang, Xiangqiang Kong, Jianlong Dai, Zhen Luo, Zhenhuai Li, Hequan Lu, Shizhen Xu, Wei Tang, Dongmei Zhang, Weijiang Li, Chengsong Xin, Hezhong Dong

**Affiliations:** 1 Cotton Research Center, Shandong Academy of Agricultural Sciences, Jinan, Shandong, China; 2 School of Life Sciences, Shandong University, Jinan, Shandong, China; 3 College of Life Science, Shandong Normal University, Jinan, Shandong, China; Estacion Experimental del Zaidin, SPAIN

## Abstract

Cotton is sensitive to waterlogging stress, which usually results in stunted growth and yield loss. To date, the molecular mechanisms underlying the responses to waterlogging in cotton remain elusive. Cotton was grown in a rain-shelter and subjected to 0 (control)-, 10-, 15- and 20-d waterlogging at flowering stage. The fourth-leaves on the main-stem from the top were sampled and immediately frozen in liquid nitrogen for physiological measurement. Global gene transcription in the leaves of 15-d waterlogged plants was analyzed by RNA-Seq. Seven hundred and ninety four genes were up-regulated and 1018 genes were down-regulated in waterlogged cotton leaves compared with non-waterlogged control. The differentially expressed genes were mainly related to photosynthesis, nitrogen metabolism, starch and sucrose metabolism, glycolysis and plant hormone signal transduction. KEGG (Kyoto Encyclopedia of Genes and Genomes) analysis indicated that most genes related to flavonoid biosynthesis, oxidative phosphorylation, amino acid metabolism and biosynthesis as well as circadian rhythm pathways were differently expressed. Waterlogging increased the expression of anaerobic fermentation related genes, such as alcohol dehydrogenase (*ADH*), but decreased the leaf chlorophyll concentration and photosynthesis by down-regulating the expression of photosynthesis related genes. Many genes related to plant hormones and transcription factors were differently expressed under waterlogging stress. Most of the ethylene related genes and ethylene-responsive factor-type transcription factors were up-regulated under water-logging stress, suggesting that ethylene may play key roles in the survival of cotton under waterlogging stress.

## Introduction

Waterlogging is a well-known abiotic stress that can severely damage crop production worldwide. It usually occurs in areas with high rainfall and/or poor drainage [1]. Low oxygen is known to be a serious factor in the adverse effects of waterlogging [2]. The low oxygen condition leads to less adenosine triphosphate (ATP) production and has negative impacts on cellular energy status. Therefore, a key feature for the acclimation to low oxygen environment is to activate genes encoding proteins and enzymes for anaerobic fermentation, glycolysis, transcription factors, and signaling pathways in order to allow biological and physiological adjustments to the low oxygen conditions [3].

For the past few years, studies in Arabidopsis (*Arabidopsis thaliana*), gray poplar (*Populus* × *canescens*), maize (*Zea mays* L.), and rice (*Oryza sativa*) revealed that low oxygen caused drastic changes in transcription, translation, and metabolite levels [1, 4–9]. Global gene expression analysis in these species has revealed complex responses to low oxygen, involving significant changes in 5–10% of all the genes assayed [10–14]. Analysis of Arabidopsis lines over- or under-expressing sucrose synthase 1 (*SUS1*) and sucrose synthase 4 (*SUS4*) or the ethanolic fermentation genes alcohol dehydrogenase (*ADH*) has indicated that these genes are essential for tolerance to low oxygen [15–18].

Waterlogging usually results in a rapid decrease in photosynthetic rate, ranging from 10 to 90% in different plant species [19]. Thus, waterlogging response of plants has been strongly associated with regulation of genes related to photosynthesis and photosystem II (PSII). The expression of Chlorophyll a-b binding protein 4 (*LHCB4*), a gene involved in the light-harvesting complex of PSII, was specifically reduced in waterlogged sesame [20]. Our previous study also showed that the expression of Gh*LHCB* was significantly down-regulated by waterlogging stress [21].

Plant hormones play important roles in regulating developmental processes and signaling networks involved in plant responses to abiotic stresses. The induction of hormones under waterlogging stress is involved in signaling cascades, including increases in ethylene [22, 23], abscisic acid (ABA) [24] and gibberellic acid (GA) [25], and a reduction in cytokinin (CK) [26] and auxin (IAA) [10]. ABA is an important signal which can be induced by some abiotic stress in regulating stomatal conductance and photosynthesis and transpiration [27]. An increase in ABA concentration was noted in leaves of flooded alfalfa [28]. Bai *et al*. [29] also found an increased ABA content in Malus leaves under hypoxia stress, indicating that ABA is a key signal in mediating responses to waterlogging.

Transcriptional factors (TFs) also play an important role in waterlogging response. Two ethylene response factors (ERFs) of Jatropha, JcERFVII-2 and JcERFVII-3, were noticeably induced in response to waterlogging [30]. The Snorkel (*SK*) and Submergence-1A (*Sub-1A*) are two pivotal genes for rice to adapt to different types of waterlogging; although both genes encode ethylene-responsive factor-type transcription factors, they function in opposite ways [31, 32]. Studies of Arabidopsis have shown that group VII ERF participated in oxygen sensing through the N-end rule pathway [33]. *HRE1*, *HRE2*, *RAP2*.*2*, and *RAP2*.*12* are four members of group-VII ERFs; overexpression of these four genes significantly improved low oxygen survival by promoting the expression of genes involved in low oxygen adaptation [34–36]. Like *ERF*, the expression of *MYB* and *WRKY* has also been regulated in plant under waterlogging stress, which activates many vital processes to cope with stress [37].

Cotton is a crop sensitive to waterlogging. In recent years, the growth response and yield loss of cotton to waterlogging stress have been extensively investigated [38–42]. Waterlogging stress decreased photosynthetic rate, stomatal conductance, and plant height, as well as biomass and lint yield [40–43]. Bange *et al*. [42] reported that intermittent waterlogging caused an approximately 38% reduction in cotton yield. Hodgson [44] showed that waterlogging stress of 4, 8, 16 and 32 h significantly decreased cotton yield. When subjected to waterlogging for 3, 6, 9 and 12 d, cotton suffered a lint yield reduction of 16, 24.1, 39.5 and 50.2%, respectively [45]. Our previous work also showed considerable yield loss due to waterlogging for 10 d, 15 d and 20 d at flowering stage [21]. Although the morphological and physiological changes including plant growth reduction and yield loss under anaerobic conditions have been well documented, limited work has been done to elucidate changes and adaptations of waterlogged cotton at the molecular level. In this study, we performed a transcriptome analysis using RNA-seq to investigate gene expression patterns in the waterlogged cotton leaves to provide new insights into the molecular responses of cotton to waterlogging under field conditions.

## Materials and methods

### Plant material and waterlogging treatment

Experiments were conducted in an electrical rain-shelter at the Experimental Station of Shandong Cotton Research Center, Linqing (115°42'E, 36°61'N), Shandong, China, in 2015. Twelve bottomless pools (3 m × 4 m) separated by concrete walls (13 cm thick and 1.5 m deep) to prevent soil water movement in different pools were established under the rain shelter. A commercial cotton (*Gossypium hirsutum* L.) cultivar, K638 developed by the Cotton Research Center, Shandong Academy of Agricultural Sciences, Jinan, was used in the experiment. Acid-delinted seeds of K638 were sown and allowed to grow in bottomless pools.

Waterlogging stress was established by over irrigation at peak flowering. The over irrigation of waterlogged plots was sustained till the water layer reach 20 cm above the soil surface for 10, 15 or 20 days. Plots with soil moisture of 60–70% under normal irrigation acted as non-waterlogging controls. The experiment was arranged into a randomized complete block design with 3 replications. The 10-, 15- and 20-d waterlogging treatment started on 15, 10 and 5 July, 2015. At the end of each waterlogging treatment, the surface water in each plot was manually removed and the soil moisture recovered to normal in a few days through evaporation.

After 10-, 15- and 20-d treatments, the fourth leaves on the main-stem from the top were harvested, frozen in liquid nitrogen and stored at -80°C for physiological measurement and RT-PCR analysis. The samples of each biological replicate were pooled from 6 plants to avoid any potential effects of position within the field. Leaves of 15-d waterlogged cotton plants were analyzed by RNA-Seq for global gene expression study.

The procedures of field management such as sowing, seedling, fertilizer application, plant pruning, pest control and chemical control were implemented according to local agronomic practices as described in Zhang *et al*. [21].

### RNA extraction and Solexa sequencing

RNA isolation Kit (Hua yue yang Biotechnology, China) was used for total RNA extraction. For Solexa sequencing, total RNA from 6 representative individual plants of each treatment was mixed into one biological replicate. Approximately 20 mg of total RNA was used. Tag libraries were prepared using the Illumina Gene Expression Sample Prep Kit, following the manufacturer’s protocol, as described in Luan *et al*. [46]. The libraries were then sequenced using an Illumina HiSeq 2500 with 50-bp single-end (SE) reads each.

### Gene annotation

The database of *G*. *hirsutum* L. genome (http://mascotton.njau.edu.cn/html/Data/Genomefhsequence/2015/04/20/16ab0945-19e9-49f7-a09e-8e956ec866bf.html) was used as a reference sequence to align and identify the sequencing reads. To map the reads to the reference, the alignments and the candidate gene identification procedure were conducted using the mapping and assembly with qualities software package [47]. The reference sequences were converted to binary FASTA format, and each Solexa read data subset (corresponding to one lane on the instrument) was transformed from Solexa FASTQ to Sanger FASTQ format. As recommended, each subset was separately mapped to the reference. These maps were then merged to form general maps for assembling the consensus sequences [48].

### Identification of differentially expressed genes (DEG) and functional analysis

The RPKM (Reads Per kb per Million reads) method was used to normalize the influence of different gene length and sequencing level on the calculation. Thus the result can be used directly for comparing the gene expression of different samples. The gene expression level was calculated by the formula [49]:

$$\mathrm{RPKM}=10^{6}C/\;(\mathrm{NL}/10^{3})$$

Where C represents the number of reads that uniquely aligned to the gene, N is the total number of reads that uniquely aligned to all genes in the specific sample, and L is number of bases of the gene. The *p*-value corresponding to differential transcript expression in two samples was determined from Audic’s algorithm [50], and False Discovery Rate (FDR) method was applied to determine the threshold of P-values in multiple tests. The DEGs were obtained after filtering using a FDR of ≤0.001 and an absolute value of log_2_ Ratio≥1.

GO enrichment analysis was performed for functional categorization of differentially expressed transcripts using agriGO software [51] and the P-values corrected by applying the FDR correction to control falsely rejected hypotheses during GO analysis. The pathway analysis was conducted using KEGG (www.genome.jp/kegg/).

### Real-time PCR (RT-PCR) analysis

To validate the results of the DGE-based analyses, the expression of 30 genes was determined using quantitative RT-PCR. The leaves from 9 representative individual plants of waterlogged and control plant were harvested and every 3 leaves were combined into one biological replicate and then extracted for total RNA. The samples used for qRT-PCR experiments and RNA-Seq analysis were not the same. Quantitative RT-PCR was performed according to the instructions provided for the Bio-Red iCycler iQ system. Each sample was run in triplicate on Bio-red IQ2 Sequence Detection System and Applied Biosystems software using first-strand cDNAs and SYBR Green PCR Master Mix. The first-strand cDNA was synthesized by Superscript II reverse transcriptase (Invitrogen) following procedures described in the manufacturer’s guidelines. Gene-specific primers were designed according to the gene sequences using the Primer Premier 5.0 (Premier Biosoft International, Palo Alto, CA) and then synthesized commercially (Shanghai Sangon Biological Engineering Technology & Services Co., Ltd., Shanghai, China). The specific primers for the selected genes and internal control gene (actin) used for qRT-PCR are listed in Table 1. The amplification of β-actin was used as an internal control to normalize all data. Thermal cycling was performed at an initial denaturation step at 95°C for 3 min followed by 40 cycles at 95°C for 10 s, at annealing temperatures of 60°C for 10 s, and at 72°C for 10 s.

**Table 1:** Primers used for qRT-PCR analysis.

Gene ID	Forward primer (5’-3’)	Reverse primer (3’-5’)
*β-actin*	GATTCCGTTGTCCAGAAGTCCT	TACGGTCTGCAATACCAGGGA
evm.TU.Gh_A01G1586	CGGTGGACGAATGGTGAAA	CACGTGTCACCTTCTTCCCTC
evm.TU.Gh_D12G1727	TACTTCACCACCTATCGCCTCT	AGTGCCACCAGATAAAGTCCA
evm.TU.Gh_D10G2385	GGTAGAGTTAGTGGAAGGAGCATC	CAGCCTGAATGCCAAAGAT
evm.TU.Gh_D06G1791	GGTGCCATTGCTGTTGAGG	AAATAGCCACCTGGGTAGAGC
evm.TU.Gh_A05G1261	TGGTGACTACGGTTGGGACA	CCAAGATGCTTTGAGCGTGTAT
evm.TU.Gh_A04G0218	TGGTGACTACGGATGGGACA	TGAGGACAACCTGGAACCC
evm.TU.Gh_Sca004917G01	CGATGTGGGATGGGTTGAA	TTTCAGACCATCCGCCAGTA
evm.TU.Gh_D10G0687	CACGAACTGCCCGACTCTTA	CAAGTGTGCTCCACCATATCAAG
evm.TU.Gh_D11G1242	CCCGAACACCGATACCCTT	CGAACATTGGGTGAAGCAG
evm.TU.Gh_A08G1649	TTTGTAAATCCGTGGTTGTGTC	GCCCTCCAATACCAATCACG
evm.TU.Gh_A11G1091	ACCTTTGAGCACAACCGAGAT	TTTAGAGCCGCCCAACCAT
evm.TU.Gh_A02G0689	GGGTTCCCAACAAAGACGA	AGAAGGGAACAGAGTGAGTGATG
evm.TU.Gh_A06G0272	GGTGGTGCCTGTCTTTCATC	CCTTCTTCAATGTCTGCCACC
evm.TU.Gh_D01G0066	TGTAGGGTCCTCTGCTGGTCT	TGTAATCCATAGGCAAGAACCAG
evm.TU.Gh_A03G0840	GTTACCAAATCTCCCGACTCTC	AGTTAGTGACGGCATCAGGAC
evm.TU.Gh_D11G0427	GATTTCATTCATCAGCACCTCC	CCAACACCTTCTGCTCCATT
evm.TU.Gh_A12G2129	GAGTGCGGGTCTGGTTAGG	AGGAGAAGCAGCAGGACGA
evm.TU.Gh_D11G2055	TGCGAATGCCGTGACAAAT	GGGGACTGGTGAAGGACGAT
evm.TU.Gh_A04G0007	CAACGCCATCTCCTTCTCA	CTTGAGAAGGCTAACATACTC
evm.TU.Gh_Sca005787G06	GGCGGCTCCCTATTCAGTG	CCTAAAGCGGTGAACCAGATA
evm.TU.Gh_D13G2037	ACAGTCTCCGTGAGCGTTTG	ACACGATGGCTTCCTGACT
evm.TU.Gh_A10G0789	ATGGGCGATGCTTGGTTAC	GAACTGAACATCCCTCCTG
evm.TU.Gh_Sca005646G04	TTGGCATTCCATTGCTGTT	CCACCCTATCCAACTTTCG
evm.TU.Gh_A03G0732	TTCCTTGTCGGCTACTGCG	CCCATCAGAGCCATCGTTA
evm.TU.Gh_A09G0840	GGTTGCTTCTATCACTGCTCGTT	AGTGCTTGCCCTGATTGTG
evm.TU.Gh_D13G2340	GAAGGACGCATTGAGAAATG	TTCGTGGTCGGAGTGTTGT
evm.TU.Gh_D01G2250	TTGCTCGCCACCACATCCA	CGTTCCATTCCCGTTTGTG
evm.TU.Gh_D10G2388	GGTCCTTGGTTCGCAGATT	AGAAGCGTGTAAGGTGGTGA
evm.TU.Gh_A03G2109	TGTGGTTCGGTTTCGGGT	TGTCCATATTTCCGCCACC
evm.TU.Gh_D01G1482	ACTGCCCAACAACAAACCC	TTGGAGCATTGTTTGTGGTTT

### Physiological measurements

Net photosynthetic rates of the fully expanded young leaf (4th leaf) on the main-stem from terminal were taken between 09:00 and 11:00 h on cloudless days, using a LI-6400 portable photosynthesis system (Li-Cor, Lincoln, NE, USA) with an integrated fluorescence chamber head (LI-COR 6400–40 Leaf Chamber Fluorometer). The photosynthesis-related parameters were as follows: PAR 1500 μmol m^−2^ s^−1^ at the leaf level; the leaf temperature of the instrument was set to 25°C; the CO_2_ concentration was 400 μmol mol^−1^; and the relative humidity inside the LI-COR leaf chamber varied between 45 and 60%. The 4th main-stem leaves of 9 randomly selected plants were sampled and washed with distilled water, and immediately frozen in liquid nitrogen and stored at −80°C for physiological measurement.

Nitric oxide was detected with the nitric oxide (NO) assay kit (Nan-jing Jiancheng Bioengineering Institute, China) according to the manufacturer’s guidelines. The content of soluble protein and soluble sugar was measured following procedures described in the manufacturer’s guidelines of their assay kits (Nan-jing Jiancheng Bioengineering Institute, China).

The 4th main-stem leaves from other 9 randomly selected plants per waterlogged plot were oven-dried for 72 h at 80°C and milled to analyze for N by Kjeldahl method.

### Statistics

Analysis of variance was performed with the function of completely randomized design using Data Processing System (DPS) [52]. Means were separated using Duncan’s multiple range tests at *p* = 5%.

## Results

### RNA-Seq analysis and identification of differentially expressed genes

RNA-Seq analysis was performed to determine the transcriptome response to waterlogging stress in cotton. We generated 11.7 and 11.4 million reads from libraries of non-waterlogged and waterlogged cotton plants, and obtained 10.9 and 10.8 million clean tags from non-waterlogged and waterlogged cotton libraries (SRA submission number: SRP095435) after the low quality tags were filtered out. The gene sequences of *G*. *hirsutum* genome were used as reference to align and identify the sequencing reads. This allowed for the mapping of approximately 90% of the distinct clean tags that passed our filters, representing more than 10.8 million reads per library (Table 2).

**Table 2:** Total number of sequencing reads obtained from each sample.

	Non-waterlogging		15 d-waterlogging	
	Read number	Ratio	Read number	Ratio
Clean Reads	11660531	100%	11357516	100%
Mapped Reads	10942343	93.84%	10834598	95.39%

Putative differentially expressed genes were finally selected depending on the expression profiles and whether: a) the average fold change between two treatment genes was more than or equal to two folds; and b) the FDR was less than 0.001. Transcriptome analysis identified 1812 DEGs with 794 up-regulated genes and 1018 down-regulated genes.

### Functional classification of differentially expressed genes

**Fig 1:**
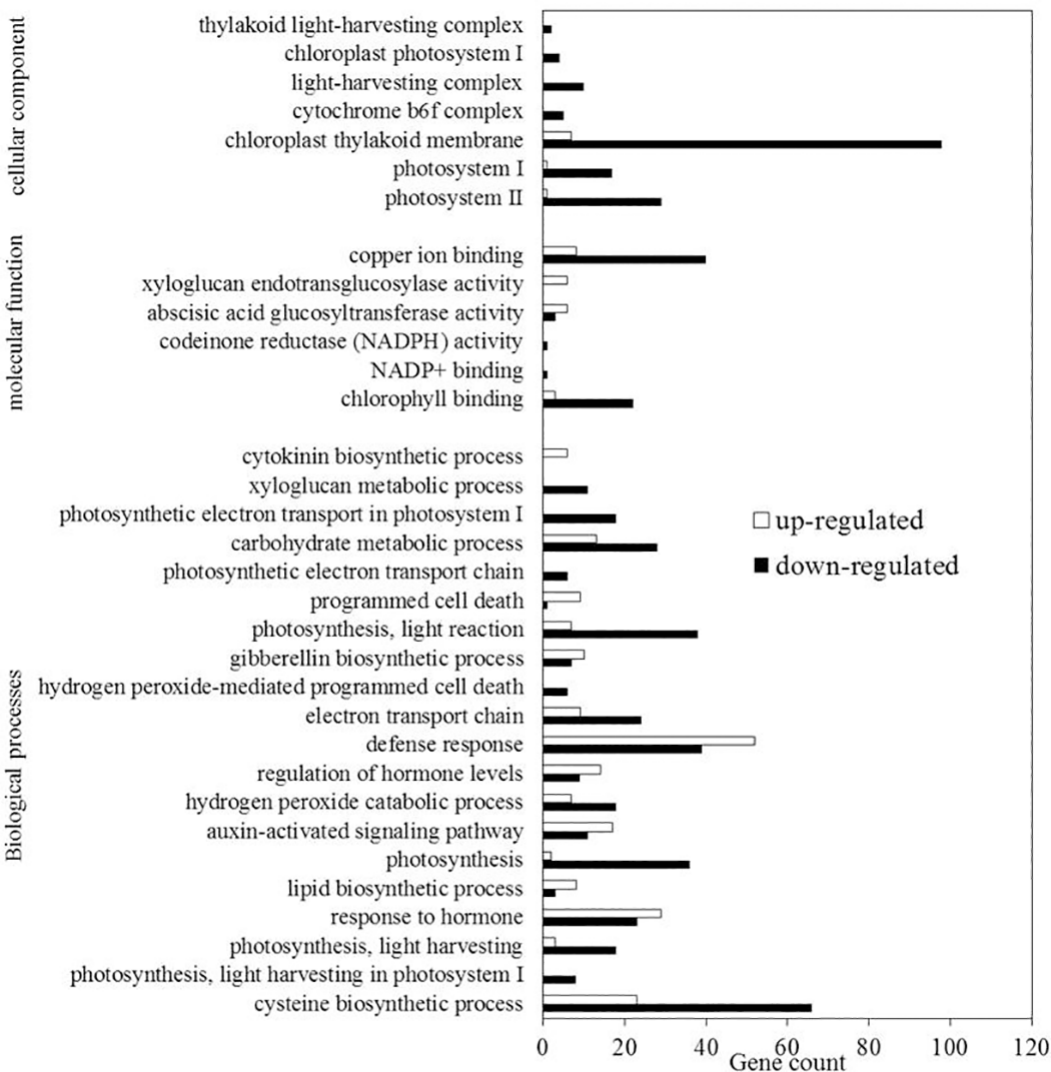
GO analysis of differentially expressed genes obtained from Solexa sequencing. The abscissa of the bar plot represents the gene count within each GO category. All processes listed had enrichment *p* values < 0.05.

Gene ontology (GO) analysis was performed by mapping each differentially expressed gene into the records of the GO database (http://www.geneontology.org/). The GO annotation of these genes is presented in Fig 1. Waterlogging stress affected a wide spectrum of physiological processes as described in the relevant GO analysis. Most of the genes related to hormone response, auxin-activated signaling pathway, regulation of hormone levels, defense response, gibberellin biosynthetic process, programmed cell death, abscisic acid glucosyltransferase activity and xyloglucan endotransglucosylase activity were up-regulated under waterlogging stress (Fig 1). On the contrary, most of the genes related to photosynthesis, light harvesting, light reaction, electron transport chain, cysteine biosynthetic process, carbohydrate metabolic process, xyloglucan metabolic process and hydrogen peroxide-mediated programmed cell death were down-regulated under waterlogging stress (Fig 1).

The GO terms were in accord with KEGG pathway analysis; e.g., enrichment of nitrogen metabolism, starch and sucrose metabolism, glycolysis and plant hormone signal transduction. In addition, significant changes in photosynthesis, flavone and flavonol biosynthesis, cysteine and methionine metabolism, steroid biosynthesis, glutathione metabolism, phenylpropanoid biosynthesis, spliceosome, as well as circadian rhythm pathways were found (Table 3). The 44 differently expressed photosynthesis related genes were all down-regulated, and 19 of 20 differently expressed antenna proteins related genes were down-regulated under waterlogging treatment. Similarly, most of the nitrogen metabolism and amino acid metabolism related DEGs were down-regulated under waterlogging treatment. However, 24 of 28 differently expressed flavonoid biosynthesis related genes were up-regulated under waterlogging (Table 3).

**Table 3:** KEGG pathway annotation of differentially expressed genes obtained from Solexa sequencing.

Pathways	DEGs with pathway annotation (347)	All genes with pathway annotation (10925)	Up-regulated gene	Down-regulated gene
Photosynthesis	44	281	0	44
Photosynthesis—antenna proteins	20	57	1	19
Flavonoid biosynthesis	28	82	24	4
Nitrogen metabolism	16	113	6	10
Phenylalanine metabolism	18	197	9	9
Stilbenoid, diarylheptanoid and gingerol biosynthesis	7	40	3	4
Phenylpropanoid biosynthesis	18	227	9	9
Circadian rhythm—plant	8	60	8	0
Phagosome	21	307	12	9
Alanine, aspartate and glutamate metabolism	12	134	3	9
Cysteine and methionine metabolism	17	232	2	15
Ether lipid metabolism	7	56	0	7
Histidine metabolism	6	50	1	5
Ascorbate and aldarate metabolism	9	110	1	8
Tyrosine metabolism	8	94	3	5
Flavone and flavonol biosynthesis	3	17	1	2
Diterpenoid biosynthesis	4	34	3	1
Glutathione metabolism	11	179	4	7
Ubiquinone and other terpenoid-quinone biosynthesis	6	73	3	3
Glycine, serine and threonine metabolism	9	137	1	8

All pathways listed had enrichment *p* values<0.05.

**Fig 2:**
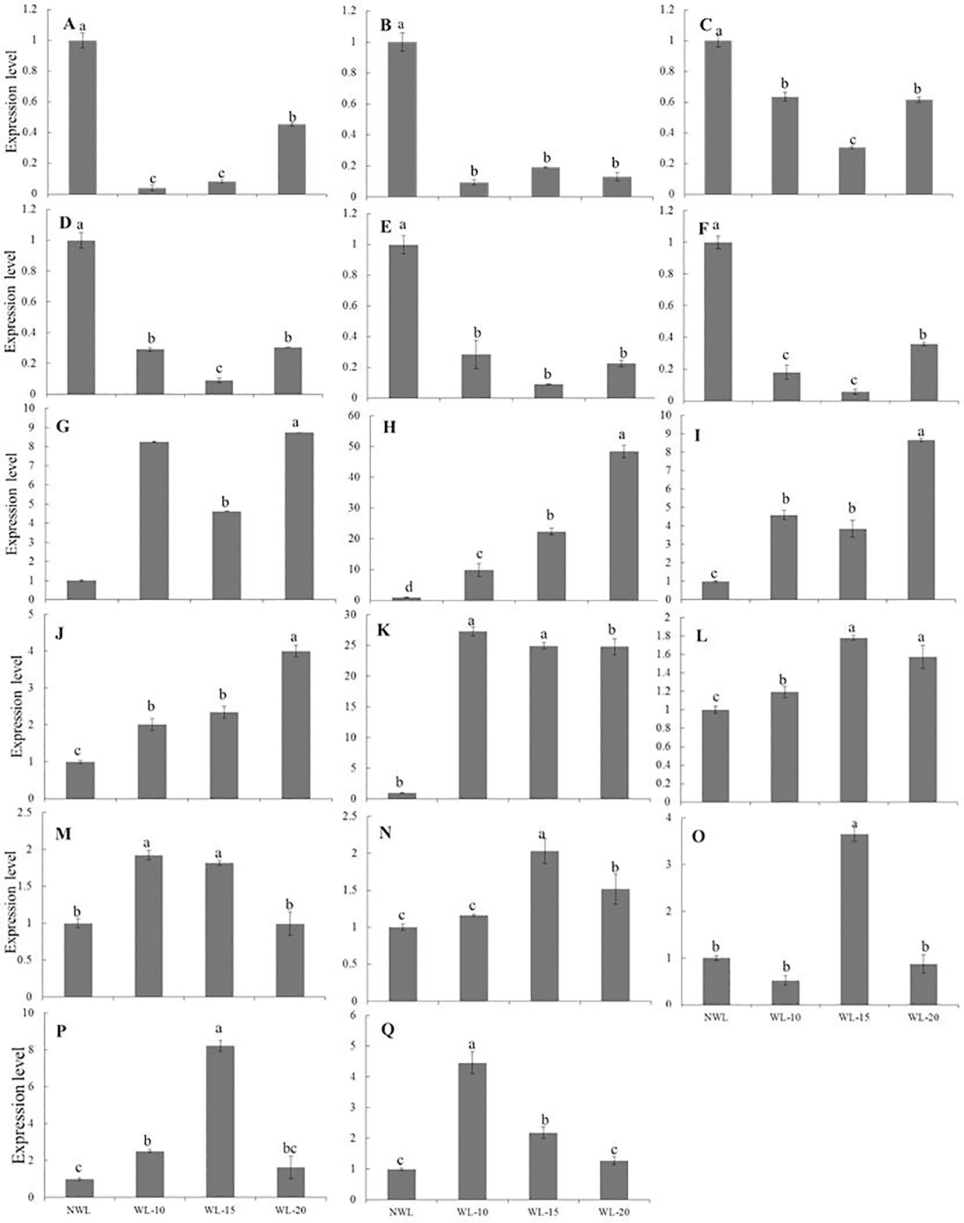
Quantitative RT-PCR of genes in cotton leaves after 10-, 15- and 20-d waterlogging treatment. A, Nitrate reductase (gene ID: evm.TU.Gh_A01G1586). B, Nitrate transporter 1.2 -like protein (gene ID: evm.TU.Gh_D12G1727). C, Chlorophyll a-b binding CP29.3 (LHCB; gene ID: evm.TU.Gh_D10G2385). D, Chlorophyll a-b binding CP29.3, chloroplastic -like protein [*Gossypium arboreum*] (LHCB; gene ID: evm.TU.Gh_D06G1791). E, Chlorophyll a-b binding protein, chloroplastic [Theobroma cacao] (LHCB; gene ID: evm.TU.Gh_A05G1261). F, Chlorophyll a-b binding 13, chloroplastic [*Gossypium arboreum*] (LHCB; gene ID: evm.TU.Gh_A04G0218); G, GA3ox1 [*Gossypium hirsutum*] (gene ID: evm.TU.Gh_D06G2009). H, GA3ox2 [*Gossypium hirsutum*] (gene ID: evm.TU.Gh_D10G0687). I, Gibberellin receptor GID1B -like protein [*Gossypium arboreum*] (gene ID: evm.TU.Gh_D11G1242). J, GID1-3 [*Gossypium hirsutum*] (gene ID: evm.TU.Gh_A08G1649). K, Gibberellin receptor GID1B -like protein [*Gossypium arboreum*] (gene ID: evm.TU.Gh_A11G1091). L, Alcohol dehydrogenase class-P -like protein [*Gossypium arboreum*] (gene ID: evm.TU.Gh_A02G0689). M, Zinc-binding alcohol dehydrogenase domain-containing 2 [*Gossypium arboreum*] (gene ID: evm.TU.Gh_D01G0066). N, AP2 domain class transcription factor [Theobroma cacao] (gene ID: evm.TU.Gh_A03G0840). O, Ethylene-responsive transcription factor [*Gossypium arboreum*] (gene ID: evm.TU.Gh_D11G0427). P, AP2/ERF domain-containing transcription factor, putative [*Theobroma cacao*] (gene ID: evm.TU.Gh_A12G2129). Q, AP2-like ethylene-responsive transcription factor ANT [*Gossypium arboreum*] (gene ID: evm.TU.Gh_D11G2055). Data are means of three biological replicates. The lower-case letter means significantly different at the P<0.05. NWL, WL-10, WL-15 and WL-20 represent 0 (non-waterlogged control)-, 10-, 15- and 20-d waterlogging at flowering, respectively.

**Fig 3:**
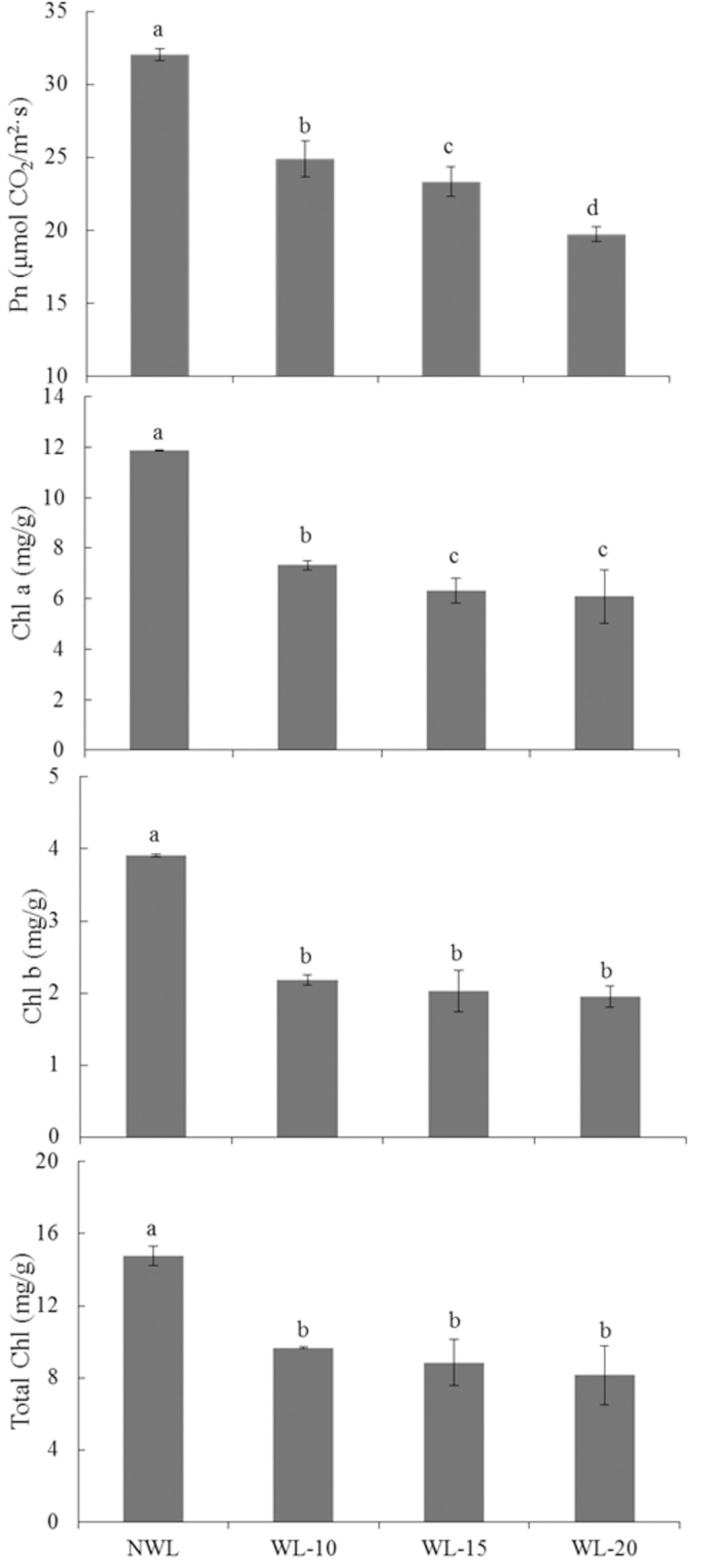
Effects of waterlogging on net photosynthetic (Pn) rate and chlorophyll (Chl) content in the main stem leaf of cotton. The lower-case letter means significantly different at the P<0.05. NWL, WL-10, WL-15 and WL-20 represent 0 (non-waterlogged control)-, 10-, 15- and 20-d waterlogging at flowering, respectively.

After 10-, 15- and 20-d waterlogging, the expression levels of the 4 *LHCB* genes were all down-regulated (Fig 2C–2F). Consistent with the decreased expression of *LHCB*; the leaf Pn rate in waterlogged cotton was reduced by 22.3, 27.2 and 38.4% after 10-, 15- and 20-d waterlogging (Fig 3). After 10-, 15- and 20-d waterlogging, the concentrations of Chl a were reduced by 38.3, 46.8 and 48.8%, Chl b by 44.1, 48.1 and 50.0%, and total Chl by 34.5, 40.1 and 44.9% (Fig 3).

### Waterlogging decreased soluble sugar contents by down-regulating the expressions of carbon metabolism related genes

Waterlogging inhibited the expression of starch and sucrose metabolism related genes. However, the expressions of glycolysis related genes were up-regulated (Table 4). The expression patterns of 2 *ADH* genes were analyzed by real-time PCR at 10-, 15- and 20-d waterlogging stress and their expression was increased by waterlogging stress (Fig 2L and 2M).

**Table 4:** Different expressed genes identified using Solexa sequencing in leaves of waterlogged cotton plants.

Functional group	Gene ID	log_2_ Ratio	*P*-value	Gene annotation
Photosynthesis	evm.TU.Gh_D04G1576	-2.33	<0.01	PsbA [Dianthus sylvestris]
	evm.TU.Gh_Sca005787G06	-2.26	<0.01	photosystem II protein D1, partial (chloroplast) [Exbucklandia populnea]
	evm.TU.Gh_A12G1101	-1.79	<0.01	[pt] photosystem II PsbC protein [Galdieria sulphuraria]
	evm.TU.Gh_A01G1598	-1.19	<0.01	Photosystem II 22 kDa, chloroplastic -like protein [Gossypium arboreum]
	evm.TU.Gh_A04G0007	-1.53	<0.01	photosystem I reaction center subunit K [Gossypium hirsutum]
	evm.TU.Gh_D05G3725	-1.20	<0.01	photosystem I reaction center subunit K [Gossypium hirsutum]
	evm.TU.Gh_D12G1385	-2.89	<0.01	Photosystem II family protein [Theobroma cacao]
	evm.TU.Gh_A03G0732	-2.02	<0.01	ATP synthase CF1 epsilon subunit [Gossypium hirsutum]
	evm.TU.Gh_D08G1218	-1.86	<0.01	cytochrome b6/f complex subunit IV [Ranunculus macranthus]
	evm.TU.Gh_Sca007622G02	-1.63	<0.01	cytochrome f [Gossypium hirsutum]
	evm.TU.Gh_D10G2385	-1.22	<0.01	Chlorophyll a-b binding CP29.3, chloroplastic -like protein [Gossypium arboreum]
	evm.TU.Gh_D06G1791	-2.00	<0.01	Chlorophyll a-b binding CP29.3, chloroplastic -like protein [Gossypium arboreum]
	evm.TU.Gh_A05G1261	-2.04	<0.01	Chlorophyll a-b binding protein, chloroplastic [Theobroma cacao]
	evm.TU.Gh_A04G0218	-1.28	<0.01	Chlorophyll a-b binding 13, chloroplastic [Gossypium arboreum]
	evm.TU.Gh_D12G1757	-1.49	<0.01	Chlorophyll a-b binding P4, chloroplastic [Gossypium arboreum]
	evm.TU.Gh_D01G0531	-2.77	<0.01	Chlorophyll a-b binding 13, chloroplastic [Gossypium arboreum]
	evm.TU.Gh_A12G1617	-1.57	<0.01	Chlorophyll a-b binding P4, chloroplastic [Gossypium arboreum]
	evm.TU.Gh_D01G1028	-1.72	<0.01	Chlorophyll a-b binding P4, chloroplastic [Gossypium arboreum]
Glycolysis	evm.TU.Gh_A05G0479	1.47	<0.01	NADP-dependent glyceraldehyde-3-phosphate dehydrogenase [Gossypium arboreum]
	evm.TU.Gh_D05G0594	1.13	<0.01	NADP-dependent glyceraldehyde-3-phosphate dehydrogenase [Gossypium arboreum]
	evm.TU.Gh_A02G0689	1.89	<0.01	Alcohol dehydrogenase class-P -like protein [Gossypium arboreum]
	evm.TU.Gh_A06G0272	2.80	<0.01	Alcohol dehydrogenase-like 1 GN = At1g22430 OS = Arabidopsis thaliana (Mouse-ear cress) PE = 2 SV = 1
	evm.TU.Gh_D04G1683	1.26	<0.01	Fructose-1,6-bisphosphatase, cytosolic [Gossypium arboreum]
Sucrose and starch metabolism	evm.TU.Gh_D13G2037	-1.23	<0.01	sucrose synthase isoform D [Gossypium hirsutum]
	evm.TU.Gh_D07G0692	-2.79	<0.01	UDP-glucose 6-dehydrogenase family protein isoform 1 [Theobroma cacao]
	evm.TU.Gh_D13G2037	-1.23	<0.01	sucrose synthase isoform D [Gossypium hirsutum]
	evm.TU.Gh_D07G0692	-2.79	<0.01	UDP-glucose 6-dehydrogenase family protein isoform 1 [Theobroma cacao]
	evm.TU.Gh_D13G2037	-1.23	<0.01	sucrose synthase isoform D [Gossypium hirsutum]
	evm.TU.Gh_A07G0152	-1.66	<0.01	Pectinesterase/pectinesterase inhibitor PPE8B [Gossypium arboreum]
	evm.TU.Gh_D05G1356	-1.85	<0.01	pectin methylesterase [Gossypium hirsutum]
Oxidative phosphorylation	evm.TU.Gh_A10G0789	-3.92	<0.01	NADH dehydrogenase subunit K [Gossypium hirsutum]
	evm.TU.Gh_Sca005646G04	-3.53	<0.01	NADH dehydrogenase subunit J [Gossypium hirsutum]
	evm.TU.Gh_A03G0732	-2.02	<0.01	ATP synthase CF1 epsilon subunit [Gossypium hirsutum]
	evm.TU.Gh_A03G0725	-1.75	<0.01	ATP synthase CF0 A subunit [Gossypium hirsutum]
	evm.TU.Gh_A03G0732	-2.02	<0.01	ATP synthase CF1 epsilon subunit [Gossypium hirsutum]
Nitrogen metabolism	evm.TU.Gh_A13G0352	-3.65	<0.01	nitrite reductase protein [Gossypium hirsutum]
	evm.TU.Gh_D13G0396	-2.66	<0.01	nitrite reductase protein [Gossypium hirsutum]
	evm.TU.Gh_D01G1872	-2.91	<0.01	Nitrate reductase [NADH] [Gossypium arboreum]
	evm.TU.Gh_A01G1586	-2.69	<0.01	Nitrate reductase [NADH] [Gossypium arboreum]
	evm.TU.Gh_A09G0840	-8.40	<0.01	asparagine synthetase [Gossypium hirsutum]
	evm.TU.Gh_D09G0861	-6.31	<0.01	asparagine synthetase [Gossypium hirsutum]
	evm.TU.Gh_D13G2340	-4.57	<0.01	asparagine synthetase [Gossypium hirsutum]
	evm.TU.Gh_D12G2422	-1.82	<0.01	NADH-dependent glutamate synthase 1 isoform 3, partial [Theobroma cacao]
Flavonoid biosynthesis	evm.TU.Gh_D08G1902	5.35	<0.01	gibberellin 3-hydroxylase 1 [Gossypium hirsutum]
	evm.TU.Gh_Sca006253G01	5.61	<0.01	chalcone synthase [Gossypium hirsutum]
	evm.TU.Gh_D12G2642	4.75	<0.01	TPA: leucoanthocyanidin reductase 2 [Gossypium raimondii]

**Fig 4:**
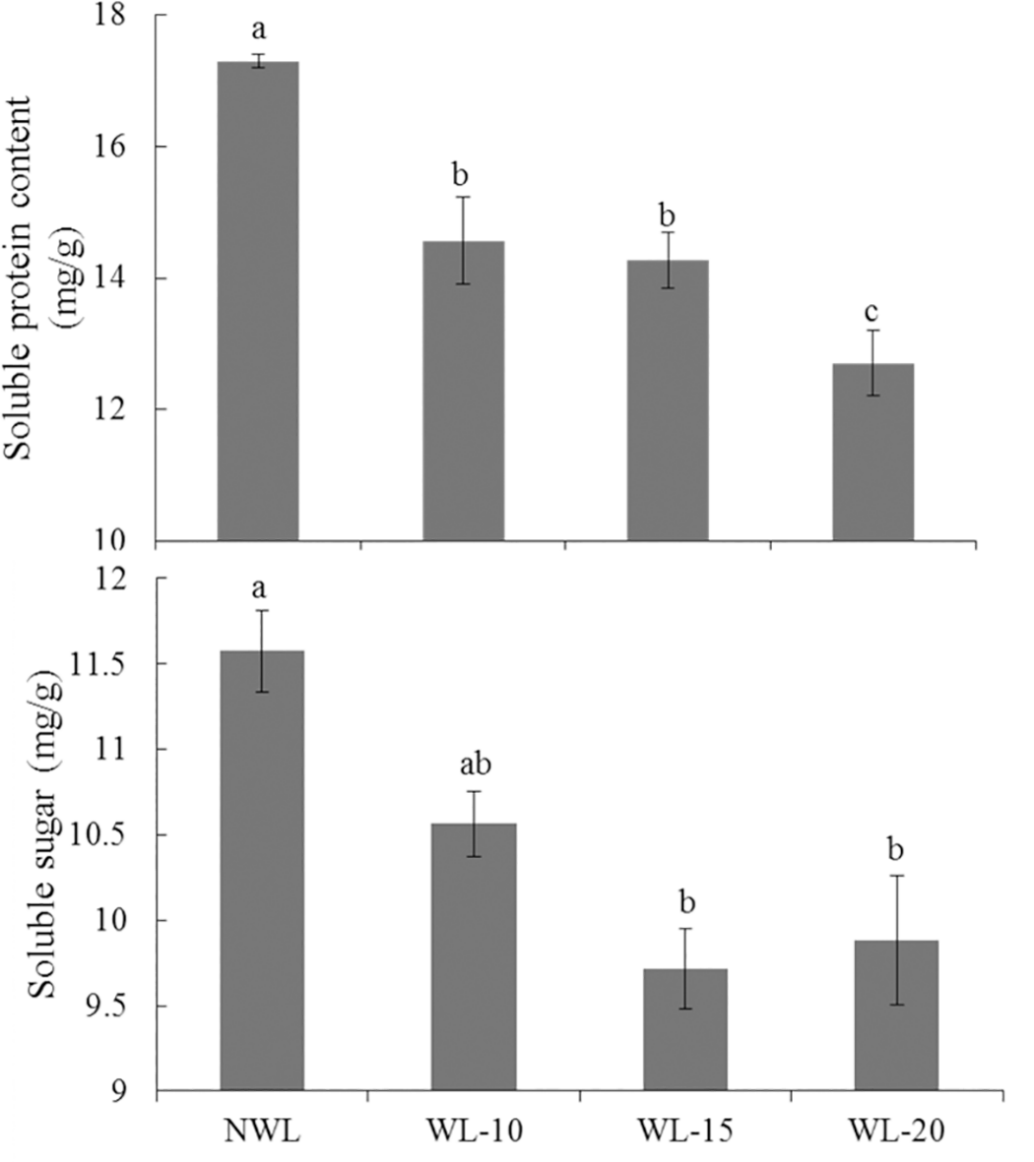
Effect of waterlogging on soluble protein and soluble sugar content of cotton leaves. The lower-case letter means significantly different at the P<0.05. NWL, WL-10, WL-15 and WL-20 represent 0 (non-waterlogged control)-, 10-, 15- and 20-d waterlogging at flowering, respectively.

The accumulation of soluble sugar in the main stem leaf was decreased by waterlogging stress, being consistent with the decreased expression of sucrose metabolism related genes. The soluble sugar content in the main stem leaves of waterlogged cotton was reduced by 8.7, 16.0 and 14.6% at 10-, 15- and 20-d waterlogging treatment, respectively (Fig 4). Similarly, the soluble protein content was decreased by 15.8, 17.5 and 26.6%, respectively (Fig 4).

### Waterlogging decreased the NO contents by down-regulating the expression of nitric oxide biosynthetic related genes

**Fig 5:**
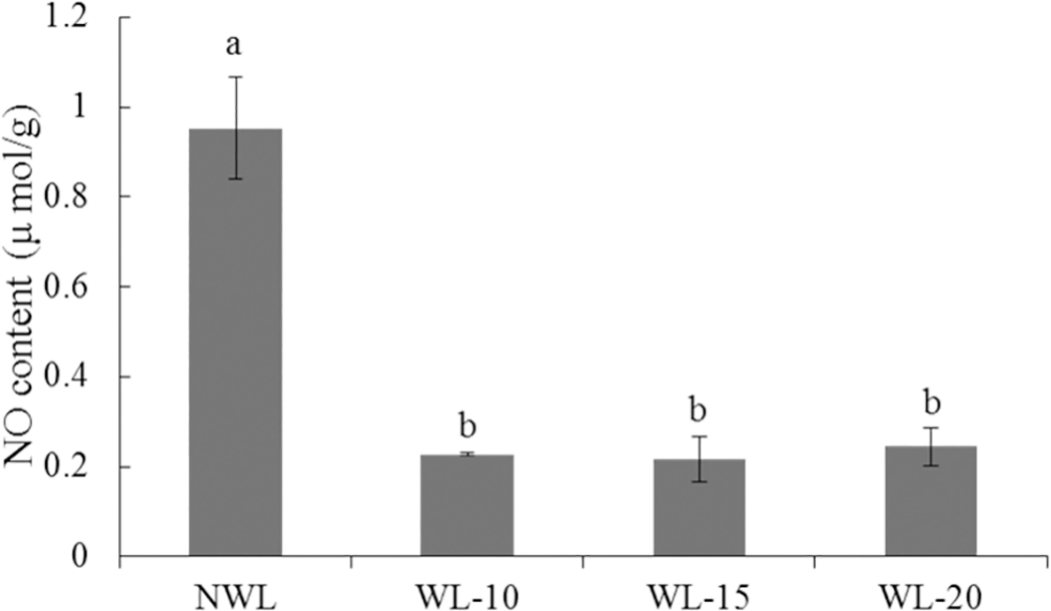
Effect of waterlogging on NO contents of cotton leaves. The lower-case letter means significantly different at the P<0.05. NWL, WL-10, WL-15 and WL-20 represent 0 (non-waterlogged control)-, 10-, 15- and 20-d waterlogging at flowering, respectively.

Many genes involved in nitric oxide biosynthetic process were down-regulated under waterlogging (Table 4). Nitrate reductase is a key enzyme in catalyzing the conversion of nitrate (NO^−3^) to nitrite (NO^-2^) and nitric oxide (NO). Nitrite reductase is responsible for the reduction of NO^−2^ to ammonium and conversion of NO^−2^ to NO. These two genes were down-regulated under waterlogging. The NO concentration in the leaf was reduced by 76.2, 77.3 and 74.4% at 10-, 15- and 20-d waterlogging treatment, respectively (Fig 5).

### Hormone related genes and transcription factor genes

There were 27 differently expressed hormone-related genes, of which 21 were up-regulated in waterlogged cotton compared with non-waterlogged control (Table 5). Compared with non-waterlogged control, the 3 JA and 2 SA related DEGs were all up-regulated, but the 3 IAA related DEGs were down-regulated in waterlogged cotton (Table 5). The 9 differently expressed GA-related genes, which include 4 GA biosynthesis genes, were all up-regulated in leaf of waterlogged cotton compared with non-waterlogged control (Table 5). Five of the 8 differently expressed ABA-related genes were up-regulated in waterlogged cotton compared with non-waterlogged control (Table 5). Interestingly, the 2 ABA biosynthesis genes, 9-cis-epoxycarotenoid dioxygenase (*NCED*), were all down-regulated, but the 2 ABA degradation genes, abscisic acid 8'-hydroxylase (*CYP707A*), were all up-regulated under waterlogging stress (Table 5).

**Table 5:** Differently expressed hormone related genes identified using Solexa sequencing in leaves of waterlogged cotton plants.

hormone	Gene ID	log_2_ Ratio	*P*-value	Gene annotation
Auxin				
	evm.TU.Gh_A06G0714	-2.37	<0.01	Auxin-induced 15A [Gossypium arboreum]
	evm.TU.Gh_D06G0824	-4.11	<0.01	Auxin-induced 15A [Gossypium arboreum]
	evm.TU.Gh_A04G0767	-1.08	<0.01	Auxin transporter-like protein 2 [Gossypium arboreum]
GA				
	evm.TU.Gh_D06G2009	2.96	<0.01	GA3ox1 [Gossypium hirsutum]
	evm.TU.Gh_Sca004917G01	3.47	<0.01	GA2ox3 [Gossypium hirsutum]
	evm.TU.Gh_D10G0687	2.49	<0.01	GA3ox2 [Gossypium hirsutum]
	evm.TU.Gh_D08G1902	5.35	<0.01	gibberellin 3-hydroxylase 1 [Gossypium hirsutum]
	evm.TU.Gh_A08G1649	2.00	<0.01	GID1-3 [Gossypium hirsutum]
	evm.TU.Gh_D11G1242	1.43	<0.01	Gibberellin receptor GID1B -like protein [Gossypium arboreum]
	evm.TU.Gh_A11G1091	1.88	<0.01	Gibberellin receptor GID1B -like protein [Gossypium arboreum]
	evm.TU.Gh_D11G2675	1.10	<0.01	GID1-5 [Gossypium hirsutum]
	evm.TU.Gh_A11G2361	1.13	<0.01	GID1-4 [Gossypium hirsutum]
Ethylene	evm.TU.Gh_A05G1492	2.19	<0.01	ACC oxidase 1 [Gossypium hirsutum]
ABA	evm.TU.Gh_D03G1182	1.85	<0.01	Abscisic acid 8&apos;-hydroxylase 2 [Theobroma cacao]
	evm.TU.Gh_A01G0280	-1.93	<0.01	Nine-cis-epoxycarotenoid dioxygenase 3 [Theobroma cacao]
	evm.TU.Gh_A12G1838	-1.88	<0.01	Nine-cis-epoxycarotenoid dioxygenase 4 [Theobroma cacao]
	evm.TU.Gh_D01G2250	2.33	<0.01	Polyketide cyclase/dehydrase and lipid transport superfamily protein [Theobroma cacao]
	evm.TU.Gh_D03G1182	1.85	<0.01	Abscisic acid 8&apos;-hydroxylase 2 [Theobroma cacao]
	evm.TU.Gh_D01G2250	2.33	<0.01	Abscisic acid receptor PYL4, Polyketide cyclase/dehydrase and lipid transport superfamily protein [Theobroma cacao]
	evm.TU.Gh_D10G2388	2.55	<0.01	Abscisic acid receptor PYL4, Polyketide cyclase/dehydrase and lipid transport superfamily protein [Theobroma cacao]
	evm.TU.Gh_D04G0015	-2.11	<0.01	Highly ABA-induced PP2C gene 3, putative [Theobroma cacao]
BR	evm.TU.Gh_A04G1027	2.45	<0.01	Cyclin-D3-1 -like protein [Gossypium arboreum]
SA	evm.TU.Gh_A08G2190	1.14	<0.01	Regulatory NPR1 -like protein [Gossypium arboreum]
	evm.TU.Gh_D02G0824	1.31	<0.01	Transcription factor TGA7 [Gossypium arboreum]
JA	evm.TU.Gh_D10G0531	1.18	<0.01	Protein TIFY 11B -like protein [Gossypium arboreum]
	evm.TU.Gh_A08G1412	1.18	<0.01	bHLH domain protein [Gossypium hirsutum]
	evm.TU.Gh_D08G1707	1.29	<0.01	bHLH domain protein [Gossypium hirsutum]

To determine if GA-related genes in leaves of waterlogged cotton were differentially regulated as described in RNA-Seq data and check their expression patterns under different duration of waterlogging stress, the expression patterns of 2 GA biosynthesis genes (*GA3ox1*, *GA3ox2*) and GA receptor genes (*GID1B*, *GID1*-3) were analyzed by real-time PCR after 10-, 15- and 20-d waterlogging stress. The expression level of *GA3ox1*, *GA3ox2*, *GID1B* and *GID1*-3 was up-regulated by waterlogging stress. The expression of these genes in cotton leaves increased greatly after waterlogging stress and most of them peaked after 20 d-waterlogging (Fig 2G–2K). Thirty-four waterlogging-regulated TFs were identified, with 26 up-regulated and 8 down-regulated (Table 6). These TFs including 13 *ERF*s, 15 *MYB*s and 6 *WRKY*s with 9 *ERF*, 13 *MYB* and 4 differentially expressed *WRKY* TFs were up-regulated by waterlogging stress. The expression level of three *AP2/ERFs* was up-regulated after 10, 15 and 20 days stress and two of them peaked after 15-d stress, the other one peaked after 10-d waterlogging stress (Fig 2N–2Q).

**Table 6:** Different expressed transcription factor genes identified using Solexa sequencing in waterlogged cotton.

Transcription factor	Gene ID	log_2_ Ratio	*P*-value	Gene annotation
ERF	evm.TU.Gh_A07G0379	2.10	<0.01	AP2/ERF domain-containing transcription factor, putative [Theobroma cacao]
	evm.TU.Gh_A08G2422	-2.19	<0.01	AP2/ERF domain-containing transcription factor, putative [Theobroma cacao]
	evm.TU.Gh_A03G0840	2.00	<0.01	AP2 domain class transcription factor [Theobroma cacao]
	evm.TU.Gh_D02G1153	1.94	<0.01	AP2 domain class transcription factor [Theobroma cacao]
	evm.TU.Gh_D11G0427	1.20	<0.01	Ethylene-responsive transcription factor [Gossypium arboreum]
	evm.TU.Gh_A03G0292	1.08	<0.01	Ethylene-responsive transcription factor RAP2-7 -like protein [Gossypium arboreum]
	evm.TU.Gh_A12G2129	-1.50	<0.01	AP2/ERF domain-containing transcription factor, putative [Theobroma cacao]
	evm.TU.Gh_A03G0292	1.08	<0.01	Ethylene-responsive transcription factor RAP2-7 -like protein [Gossypium arboreum]
	evm.TU.Gh_D13G1806	-1.84	<0.01	Ethylene-responsive transcription factor [Theobroma cacao]
	evm.TU.Gh_D11G2055	4.16	<0.01	AP2-like ethylene-responsive transcription factor ANT [Gossypium arboreum]
	evm.TU.Gh_D11G0427	1.20	<0.01	Ethylene-responsive transcription factor [Gossypium arboreum]
	evm.TU.Gh_A10G1483	2.03	<0.01	Ethylene-responsive transcription factor RAP2-3 -like protein [Gossypium arboreum]
	evm.TU.Gh_A13G0468	-1.07	<0.01	Ethylene-responsive transcription factor ERF119
WRKY	evm.TU.Gh_D05G1432	1.38	<0.01	WRKY transcription factor 74 [Gossypium hirsutum]
	evm.TU.Gh_D07G0318	-3.52	<0.01	WRKY52 [Gossypium aridum]
	evm.TU.Gh_A03G2109	4.31	<0.01	WRKY transcription factor 33 [Gossypium hirsutum]
	evm.TU.Gh_D05G1432	1.38	<0.01	WRKY transcription factor 74 [Gossypium hirsutum]
	evm.TU.Gh_A03G2109	4.31	<0.01	WRKY transcription factor 33 [Gossypium hirsutum]
	evm.TU.Gh_D07G0318	-3.52	<0.01	WRKY52 [Gossypium aridum]
MYB	evm.TU.Gh_A13G0668	2.27	<0.01	Myb family transcription factor APL [Gossypium arboreum]
	evm.TU.Gh_D04G1244	1.08	<0.01	Transcription factor MYB1R1 [Gossypium arboreum]
	evm.TU.Gh_A10G1496	1.58	<0.01	Myb domain protein 111, putative [Theobroma cacao]
	evm.TU.Gh_D13G0783	1.41	<0.01	Myb family transcription factor APL [Gossypium arboreum]
	evm.TU.Gh_A11G2875	1.47	<0.01	Transcription factor MYB1R1 [Gossypium arboreum]
	evm.TU.Gh_D06G0713	1.07	<0.01	Myb-like transcription factor family protein, putative [Theobroma cacao]
	evm.TU.Gh_D01G1482	2.99	<0.01	R2R3 MYB C2 repressor motif-like1 protein [Theobroma cacao]
	evm.TU.Gh_D01G2210	1.08	<0.01	Transcription factor MYB1R1 [Gossypium arboreum]
	evm.TU.Gh_D12G1140	1.82	<0.01	Transcription repressor MYB5 -like protein [Gossypium arboreum]
	evm.TU.Gh_D08G0391	3.27	<0.01	Transcription factor MYB3 -like protein [Gossypium arboreum]
	evm.TU.Gh_A01G1949	1.10	<0.01	Transcription factor MYB1R1 [Gossypium arboreum]
	evm.TU.Gh_A10G1002	-3.12	<0.01	Myb domain protein 13, putative [Theobroma cacao]
	evm.TU.Gh_A11G2726	1.52	<0.01	MYB-like protein 2 [Gossypium barbadense]
	evm.TU.Gh_A07G0140	2.90	<0.01	Transcription factor MYB3 -like protein [Gossypium arboreum]
	evm.TU.Gh_A12G1550	-1.70	<0.01	Myb-like transcription factor family protein, putative isoform 1 [Theobroma cacao]

### Confirmation of Solexa expression patterns by RT-PCR analysis

**Fig 6:**
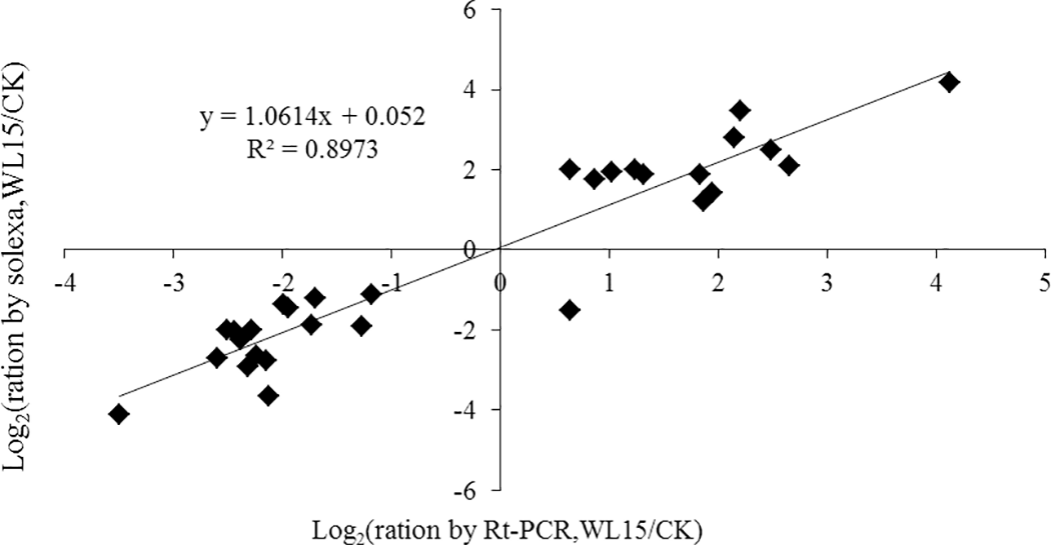
Comparison of the expression ratios of some selected genes using Solexa sequencing and qRT-PCR.

To test the reliability of Solexa sequencing, RT-PCR analysis was performed with specific primers for 30 genes, which have been identified by Solexa sequencing in which 13 genes were up-regulated and 17 genes were down-regulated. The results showed that 28 of the 30 genes had the same expression profiles as the original Solexa sequencing, and the original Solexa pattern was validated in 93.3% of the cases. This was not the case for other genes presumably because of the mutations within the primer sites or because the RNA used for Solexa sequencing and qRT-PCR were extracted from different plants. The expression patterns of the 30 genes were highly consistent with the Solexa sequencing ratios, with a relative R^2^ of 0.8973 (Fig 6).

## Discussion

Waterlogging has become one of the serious problems limiting cotton yield in recent years [43, 44]. Understanding its effects and the mechanisms would help minimize yield loss. Our previous study showed that waterlogging at flowering stage significantly inhibited plant growth and reduced biological and economical yield of cotton due to reductions in net photosynthetic rate, leaf area, boll density and boll weight [53]. In this study, sequencing method was employed to compare differential gene expression profiles of cotton leaves subjected to 15-d waterlogging stress.

### Global gene transcription changes in waterlogged cotton leaf

Waterlogging stress increased the expression of genes in glycolysis and some catabolism pathways, but reduced the expression of synthesis pathways and oxidative phosphorylation genes in poplar and maize [5, 8]. The down-regulation of these energy-related and O_2_-consuming metabolic pathways suggested that plants initiate several responses to alleviate the impact of low O_2_ during waterlogging periods. Our data showed that many genes with potential roles in carbohydrate metabolism, nitrogen metabolism and photosynthesis were down-regulated; in ethylene synthesis and perception, regulation of transcription and glycolysis were significantly up-regulated in cotton under waterlogging stress.

Waterlogging stress decreases leaf photosynthesis, and many genes involved in the photosynthesis pathway were down-regulated [54, 55]. Thus plant growth decreased with waterlogging stress due to decreasing photosynthetic rate following induced damage to cellular and photosynthetic machinery [56]. Our data showed that many genes involved in photosynthesis were down-regulated in leaves under waterlogging. Most of the genes related to chlorophyll and light-harvesting complex were down-regulated in leaves under waterlogging. Remarkably, 4 chlorophyll a/b-binding (*LHCB*s) genes which involved in the light-harvesting complex of photosystem II (PSII) were significantly down-regulated by waterlogging stress (Fig 2C–2F). The decreased plant growth under waterlogging stress may be due to the suppressed expression of photosynthesis and metabolism related genes.

### Carbon and energy metabolism as affected by waterlogging

Plants subjected to waterlogging stress shift their metabolism from oxidative phosphorylation to anaerobic fermentation to maintain ATP production by down-regulating storage metabolism, changing Suc synthase to Suc hydrolysis, and inhibiting mitochondrial respiration [3, 57–58]. Under waterlogging conditions, O_2_ limits oxidative phosphorylation and plant cells must alternate metabolic pathways to produce ATP. Plant ethanolic fermentation is activated under low-oxygen stress: PDC firstly converts pyruvate to acetaldehyde, and then ADH converts acetaldehyde to ethanol. The expression of *PDC1*, *PDC2* and *PDC4* is strongly up-regulated under waterlogging in rice, which improves the tolerance under long-term anoxia [59]. Hypoxia and anoxia induced significantly the expression of *Arabidopsis PDC1*and *PDC2*, and these two genes play key roles in the tolerance of submergence by mutant and transgenic experiments [60].

*ADH* is an important enzyme involved in ethanolic fermentation and induction of ADH during anaerobiosis has been observed in many plant species such as mungbean [61], pigeonpea [62] and rice [63]. ADH activity has been reported to increase under anoxia in all parts of plants such as roots [64], shoots [65, 66], seedlings [67] and coleoptile [68]. The activity of ADH in flood-tolerant sorghum (*Sorghum Bicolor* L.) cultivar SSG-59-3 was significantly higher than the sensitive variety S-308 [69]. The maize mutant which is deficient in one of its *ADH* genes, was more sensitive to flooding injury and died earlier than wild type plants [70]. In addition, transgenic lines over-expressing *ADH* produced significantly more ethanol than wild type plants, indicating an increase in ethanol fermentation [71]. In the present study, the expression of *ADH* was specifically enhanced in waterlogged cotton, which was parallel to our previous observation that greater activity of ADH occurred in waterlogged cotton, suggesting that *ADH* may have important roles in maintaining cotton growth under waterlogging stress. In addition, many genes involved in the synthesis of sucrose and starch were down-regulated (Table 4) in waterlogged cotton, which may be the reason for the reduction in total soluble sugar content (Fig 4).

### Nitrogen metabolism as affected by waterlogging

**Fig 7:**
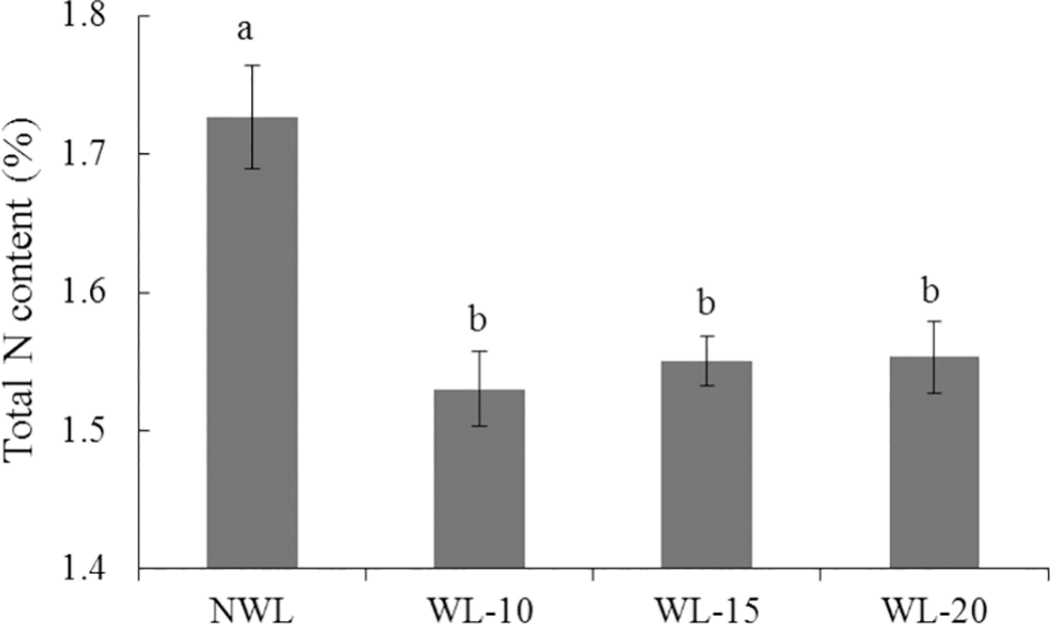
Effect of waterlogging on the total N content of cotton leaves. The lower-case letter means significantly different at the P<0.05. NWL, WL-10, WL-15 and WL-20 represent 0 (non-waterlogged control)-, 10-, 15- and 20-d waterlogging at flowering, respectively.

Waterlogging results in root anoxia and increased denitrification, leading to significant N loss from soil and potential nitrous oxide (N(_2_)O) emissions [72]. Plant repressed nutrient uptake and inhibited biosynthetic processes in order to avoid the occurrence of complete anoxia under waterlogging stress [5]. In this study, the soluble protein and N contents (Fig 7) in the leaves of waterlogged cotton were reduced, which might be due to the increased expression of nitrogen metabolism related genes and decreased expression of amino acid biosynthesis related genes.

Nitric oxide (NO) is an essential endogenous signal molecule involved in multiple physiological processes in plants [73]. It can also act as a secondary messenger in environmental stress signal transduction [74, 75]. It was reported that NO could increase the photosynthetic pigments and net photosynthesis rate of leaves [76]. It could facilitate anaerobic survival of plants [77–79]. Chen [80] and Song [81] showed that providing NO by spraying SNP (sodium nitroprusside), a NO donor, alleviated the damage to plants caused by waterlogging stress. It has been observed that long periods of low oxygen survival can be achieved when NO^−3^ is provided because NO can be synthesized from NO^−3^ via NR and NiR [77, 82–83]. In this study, the NO content in the waterlogged cotton leaves decreased (Fig 5), possibly due to the decreased expression of *NR* and *NiR* under waterlogging stress.

### Hormone responses to waterlogging

Ethylene is considered to be the first warning signal in plants under hypoxia stress. Ethylene concentration increased under waterlogging stress to stimulate shoot elongation in wetland plants and it is likely to be a key player in the interactions among hormones like ABA and GA [84–88]. In this study, the ACC oxidase 1 (*ACO1*), a gene involved in ethylene synthesis, was up-regulated in cotton leaves (Table 5). Furthermore, 9 of the 13 differently expressed *ERF* TFs were up-regulated under waterlogging stress, suggesting that ethylene may play an essential role in waterlogging response of cotton.

Gibberellin has been reported to enhance waterlogging tolerance in rice and *Rumex palustris* under submerged condition [89, 90]. GA could induce the elongation of internode to bring out rice leaves from water surface for aerobic respiration [3, 32, 86, 91]. GA is perceived by its nuclear receptors GA INSENSITIVE DWARF1s (GID1s), which then trigger degradation of downstream repressors DELLAs [92]. In the present study, the expression of GA biosynthesis genes and 3 *GID1* genes were up-regulated by waterlogging stress. It is suggested that GA may have positive role in maintaining cotton growth under waterlogging stress.

IAA plays important roles in plant growth and development [93]. In the present study, 3 auxin-related genes were down-regulated in the leaves of waterlogged cotton (Table 4). Interestingly, changes in the transcript levels of these genes were frequently associated with changes in the IAA content in leaves of 15-d waterlogged cotton plant in our previous report [21]. Besides, plant hormones JA, SA and BR are well regulators of plant growth. Nguyen *et al*. [94] reported that they are possibly involved in a network of signaling cascades to help plants adapt to abiotic stresses. In this study, many differently expressed JA, SA and BR related genes were up-regulated in waterlogged cotton, indicating that JA, SA and BR are possibly involved in waterlogging response (Table 5).

Many TFs are differentially regulated under waterlogging stress and have important roles in plant response to waterlogging stress. Thirty- four differentially regulated TFs were found in our experiment, suggesting that transcriptional regulation plays a key role in the waterlogging response in cotton. *HRE* and *RAP* type *ERF* genes were shown to be the important regulators in plant responses to hypoxia and anoxia [33–36, 95]. A more recent study showed that the N-end rule pathway of protein degradation acts as a homeostatic sensor of hypoxia in Arabidopsis through the regulation of key hypoxia-response TFs [33–34, 36]. In addition, Licausi *et al*. [96] identified two Arabidopsis hypoxia-inducible *ERF* genes, *HRE1* and *HRE2*, which could induce anaerobic gene expression and ethanol fermentation to enhancing plant tolerance of anaerobic stress. The Arabidopsis *RAP2*.*2* and rice *SUB1A*, which play important roles in survival under hypoxia can be induced by *ERF* [32, 34]. In this study, 13 differently expressed ERFs were identified and 9 of them were up-regulated under waterlogging stress (Table 6). The increased expression of *ERF* may have important functions in enhancing plant tolerance of anaerobic stress through inducing some waterlogging tolerance related genes. A further analysis of waterlogging-induced TFs seems required to find more functional candidate genes involved in the adaption to waterlogging stress.

As mentioned above, several published studies of other plant species have provided some fundamental clues to plant response to waterlogging, which identified a common response including the up-regulation of ethanolic fermentative genes and the down-regulation of genes involved in photosynthesis, electron transport chain, and light harvesting. In the present study, cotton showed the up-regulation of genes involved in ethylene production and signaling which was commonly found in response to low oxygen in other plant species. And the alteration of carbohydrate metabolism and the up-regulation of *AP2/ERF* genes appeared to be a common response to low oxygen stress in plants. Although, plants response to waterlogging had so much in common, various processes were specifically regulated in each species. Besides, cotton is a species with indeterminate growth habit and large compensative ability after the removal of stress, which makes cotton different from other crops. For example, four *AP2/ERF* genes were up-regulated in waterlogged cotton, as shown in the present results (Fig 2). Although this result was consistent with the up-regulation of two Arabidopsis *ERF* genes, *HRE1* and *HRE2*, in hypoxia conditions [96], the extent in cotton was far less than Arabidopsis. It indicates that ethylene could play a more important role in the hypoxia response of Arabidopsis than cotton. Additionally, contrary to what has been observed in submerged Jatropha [30], the expression of *NR* was down-regulated in waterlogged cotton in our experiment suggesting the different-regulation of the *NR* gene may be species-specific responses to waterlogging stress. Taken together, we propose that the difference between cotton and other plants may also be attributed to the duration or the depth of waterlogging stress in the present study.

### Conclusions

**Fig 8:**
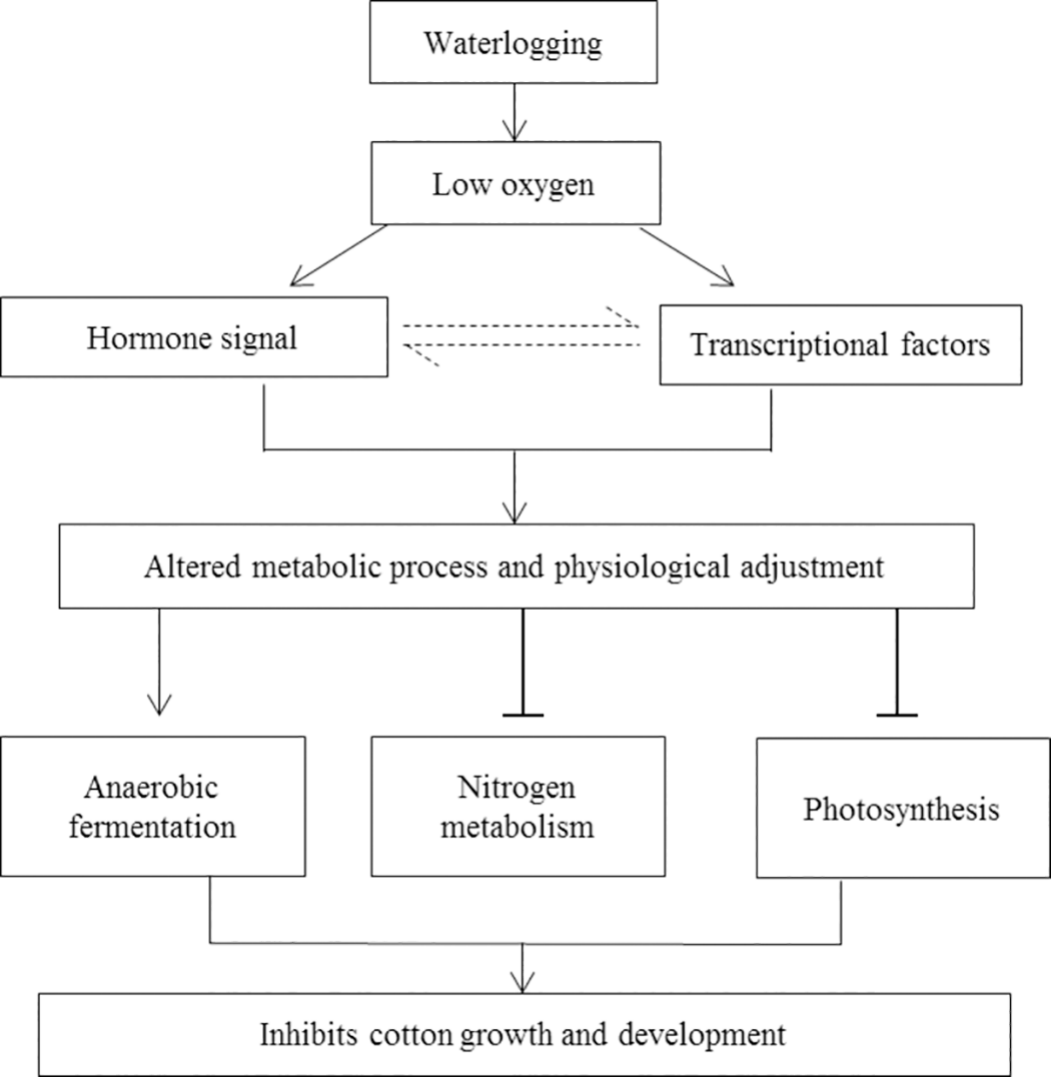
Simplified overview of cotton response to waterlogging.

Conclusively, waterlogging stress resulted in a number of biological change such as reduced photosynthesis and decreased contents of chlorophyll, NO, total soluble sugar and protein. These biological changes in waterlogged cotton were attributed to the decreased expression of photosynthesis related genes, and increased expression of glycolytic pathway and fermentation genes. Furthermore, the changes in the expression pattern of these genes might be regulated by synthesis and perception of plant hormones like ethylene and GA, and some transcription factors like ERF, MYB and WRKY (Fig 8).

## References

[1] Bailey-Serres J, Voesenek LA. Life in the balance: a signaling network controlling survival of flooding. Curr. Opin. Plant Biol. 2010; 13 (5): 489–494. doi: 10.1016/j.pbi.2010.08.002 20813578 10.1016/j.pbi.2010.08.002

[2] Jackson MB, Davies DD, Lambers H. Plant life under oxygen deprivation: ecology, physiology and biochemistry. Vegetation. 1992; 103 (2): 167–168.

[3] Bailey-Serres J, Fukao T, Gibbs DJ, Holdsworth MJ, Lee SC, Licausi F, et al Making sense of low oxygen sensing. Trends Plant Sci. 2012; 17: 129–138. doi: 10.1016/j.tplants.2011.12.004 22280796 10.1016/j.tplants.2011.12.004

[4] Branco-Price C, Kaiser KA, Jang CJ, Larive CK, Bailey-Serres J. Selective mRNA translation coordinates energetic and metabolic adjustments to cellular oxygen deprivation and reoxygenation in *Arabidopsis thaliana*. Plant J. 2008; 56: 743–755. doi: 10.1111/j.1365-313X.2008.03642.x 18665916 10.1111/j.1365-313X.2008.03642.x

[5] Jürgen K, Jost H, Howell KA, Adam C, Heinz R, A Harvey M, et al Differential response of gray poplar leaves and roots underpins stress adaptation during hypoxia, Plant Physiol. 2009; 149: 461–473. doi: 10.1104/pp.108.125989 19005089 10.1104/pp.108.125989 PMC2613732

[6] Mustroph A, Zanetti ME, Jang CJ, Holtan HE, Repetti PP, Galbraith DW, et al Profiling translatomes of discrete cell populations resolves altered cellular priorities during hypoxia in Arabidopsis. Proc.Natl.Acad.Sci. U.S.A. 2009; 106: 18843–18848. doi: 10.1073/pnas.0906131106 19843695 10.1073/pnas.0906131106 PMC2764735

[7] Narsai R, Howell KA, Carroll A, Ivanova A, Millar AH, Whelan J. Defining core metabolic and transcriptomic responses to oxygen availability in rice embryos and young seedlings. Plant Physiol. 2009; 151:306–322. doi: 10.1104/pp.109.142026 19571305 10.1104/pp.109.142026 PMC2736006

[8] Thirunavukkarasu N, Hossain F, Mohan S, Shiriga K, Mittal S, Sharma R, et al Genome-wide expression of transcriptomes and their co-expression pattern in subtropical maize (*Zea mays* L.) under waterlogging stress. PLoS ONE. 2013; 8: e70433 doi: 10.1371/journal.pone.0070433 23936429 10.1371/journal.pone.0070433 PMC3735631

[9] Lee SC, Mustroph A, Sasidharan R, Vashisht D, Pedersen O, Oosumi T, et al Molecular characterization of the submergence response of the Arabidopsis thaliana ecotype Columbia. New.Phytol. 2011; 190:457–471. doi: 10.1111/j.1469-8137.2010.03590.x 21231933 10.1111/j.1469-8137.2010.03590.x

[10] Klok EJ, Wilson IW, Wilson D, Chapman SC, Peacock WJ, Dolferus R, et al Expression profile analysis of the low-oxygen response in Arabidopsis root cultures. Plant Cell. 2002; 14: 2481–2494. doi: 10.1105/tpc.004747 12368499 10.1105/tpc.004747 PMC151230

[11] Liu F, Vantoai T, Moy LP, Bock G, Linford LD, Quackenbush J. Global transcription profiling reveals comprehensive insights into hypoxic response in Arabidopsis. Plant Physiol. 2005; 137: 1115–1129. doi: 10.1104/pp.104.055475 15734912 10.1104/pp.104.055475 PMC1065411

[12] Loreti E, Poggi A, Novi G, Alpi A, Perata P. A genome-wide analysis of the effects of sucrose on gene expression in Arabidopsis seedlings under anoxia. Plant Physiol. 2005; 137(3):1130–8. doi: 10.1104/pp.104.057299 15734908 10.1104/pp.104.057299 PMC1065412

[13] Lasanthi-Kudahettige R, Magneschi L, Loreti E, Gonzali S, Licausi F, Novi G, et al Transcript profiling of the anoxic rice coleoptile. Plant Physiol. 2007; 144(1): 218–31. doi: 10.1104/pp.106.093997 17369434 10.1104/pp.106.093997 PMC1913783

[14] Kreuzwieser J, Hauberg J, Howell KA, Carroll A, Rennenberg H, Millar AH, et al Differential response of gray poplar leaves and roots underpins stress adaptation during hypoxia. Plant Physiol. 2009; 149(1): 461–73. doi: 10.1104/pp.108.125989 19005089 10.1104/pp.108.125989 PMC2613732

[15] Ellis MH, Dennis ES, Peacock WJ. Arabidopsis roots and shoots have different mechanisms for hypoxic stress tolerance. Plant Physiol. 1999; 119; 57–64. 9880346 10.1104/pp.119.1.57 PMC32242

[16] Ismond KP, Dolferus R, De Pauw M, Dennis ES, Good AG. Enhanced low oxygen survival in Arabidopsis through increased metabolic flux in the fermentative pathway, Plant Physiol. 2003; 132: 1292–1302. doi: 10.1104/pp.103.022244 12857811 10.1104/pp.103.022244 PMC167069

[17] Kürsteiner O, Dupuis I, Kuhlemeier C. The Pyruvate decarboxylase1 gene of Arabidopsis is required during anoxia but not other environmental stresses. Plant Physiol. 2003; 132 (2):968–978. doi: 10.1104/pp.102.016907 12805625 10.1104/pp.102.016907 PMC167035

[18] Bieniawska Z. Functional analysis of the sucrose synthase gene family in Arabidopsis thaliana. D. Sc. Dissertation, University of Potsdam. 2007. 10.1111/j.1365-313X.2006.03011.x 17257168

[19] Kozlowski TT, Pallardy SG. Effects of flooding on metabolism In: Kozlowski TT, editor. Flooding and plant growth. Orlando, FL: Academic Press; 1984 pp. 165–193.

[20] Wang L, Zhang Y, Qi X, Li D. Global gene expression responses to waterlogging in roots of sesame (*Sesamum indicum* L.). Acta Physiologiae Plantarum. 2012; 34(6): 2241–2249.

[21] Zhang Y, Chen Y, Lu H, Kong X, Dai J, Li Z, et al Growth, lint yield and changes in physiological attributes of cotton under temporal waterlogging. Field Crops Research. 2016; 194: 83–93.

[22] Barnawal D, Bharti N, Maji D, Chanotiya CS, Kalra A. 1-Aminocyclopropane-1-carboxylic acid (ACC) deaminase-containing rhizobacteria protect Ocimum sanctum plants during waterlogging stress via reduced ethylene generation. Plant Physiol. Biochem. 2012; 58: 227–235. doi: 10.1016/j.plaphy.2012.07.008 22846334 10.1016/j.plaphy.2012.07.008

[23] Hess N, Klode M, Anders M, Sauter M. The hypoxia responsive transcription factor genes ERF71/HRE2 and ERF73/HRE1 of Arabidopsis are differentially regulated by ethylene. Physiol. Plant. 2011; 143: 41–49. doi: 10.1111/j.1399-3054.2011.01486.x 21615413 10.1111/j.1399-3054.2011.01486.x

[24] Yang SH, Choi D. Characterization of genes encoding ABA 8'-hydroxylase in ethylene-induced stem growth of deepwater rice (*Oryza sativa* L.). Biochem. Biophys. Res. Commun. 2006; 350: 685–690. doi: 10.1016/j.bbrc.2006.09.098 17022939 10.1016/j.bbrc.2006.09.098

[25] He CJ, Morgan PW, Drew MC. Transduction of an ethylene signal is required for cell death and lysis in the root cortex of maize during aerenchyma formation induced by hypoxia. Plant Physiol. 1996; 112: 463–472. 12226403 10.1104/pp.112.2.463 PMC157969

[26] Cheng Y, Gu M, Cong Y, Zou C, Zhang X, Wang H. Combining ability and genetic effects of germination traits of *Brassica napus* L. under waterlogging stress condition. Agric. Sci. China. 2010; 9: 101–105.

[27] Ahmed S, Nawata E, Sakuratani T. Changes of endogenous ABA and ACC,and their correlations to photosynthesis and water relations in mungbean (*Vignaradiate* L. Wilczak cv. KPS1) during waterlogging. Environ. Exp. Bot. 2006; 57: 278–284.

[28] Castonguay Y, Nadeau P, Simard RR. Effects of flooding on carbohydrate and ABA levels in roots and shoots of alfalfa. Plant Cell & Environment. 1993; 16(6):695–702.

[29] Bai T, Yin R, Li C, Ma F, Yue Z, Shu H. Comparative analysis of endogenous hormones in leaves and roots of two contrasting malus species in response to hypoxia stress. Journal of Plant Growth Regulation. 2011; 30(2):119–127.

[30] Juntawong P, Sirikhachornkit A, Pimjan R, Sangsrakru D, Yoocha T, Tangphatsornruang S, et al Elucidation of the molecular responses to waterlogging in Jatropha roots by transcriptome profiling. Frontiers in Plant Science. 2014; 5(658): 658–658. 25520726 10.3389/fpls.2014.00658 PMC4251292

[31] Hattori Y, Nagai K, Furukawa S, Song XJ, Kawano R, Sakakibara H, et al The ethylene response factors SNORKEL1 and SNORKEL2 allow rice to adapt to deep water. Nature. 2009; 460: 1026–1030. doi: 10.1038/nature08258 19693083 10.1038/nature08258

[32] Xu K, Xu X, Fukao T, Canlas P, Maghirang-Rodriguez R, Heuer S, et al Sub1A is an ethylene-response-factor-like gene that confers submergence tolerance to rice. Nature. 2006; 442: 705–708. doi: 10.1038/nature04920 16900200 10.1038/nature04920

[33] Sasidharan R, Mustroph A. Plant oxygen sensing is mediated by the N-end rule pathway: A milestone in plant anaerobiosis. Plant Cell. 2011; 23: 4173–4183. doi: 10.1105/tpc.111.093880 22207573 10.1105/tpc.111.093880 PMC3269858

[34] Hinz M, Wilson IW, Yang J, Buerstenbinder K, Llewellyn D, Dennis ES, et al Arabidopsis RAP2.2: an ethylene response transcription factor that is important for hypoxia survival. Plant Physiol. 2010; 153(2): 757–72. doi: 10.1104/pp.110.155077 20357136 10.1104/pp.110.155077 PMC2879770

[35] Gibbs DJ, Lee SC, Isa NM, Gramuglia S, Fukao T, Bassel GW, et al Homeostatic response to hypoxia is regulated by the N-end rule pathway in plants. Nature. 2011; 479: 415–418. doi: 10.1038/nature10534 22020279 10.1038/nature10534 PMC3223408

[36] Licausi F, Kosmacz M, Weits DA, Giuntoli B, Giorgi FM, Voesenek LA, et al Oxygen sensing in plants is mediated by an N-end rule pathway for protein destabilization. Nature. 2011; 479: 419–422. doi: 10.1038/nature10536 22020282 10.1038/nature10536

[37] Chen W, Yao Q, Patil GB, Agarwal G, Deshmukh RK, Lin L, et al Identification and comparative analysis of differential gene expression in soybean leaf tissue under drought and flooding stress revealed by RNA-Seq. Frontiers in Plant Science. 2016; 7(244). 10.3389/fpls.2016.01044 PMC4950259 27486466

[38] Milroy SP, Bange MP. Reduction in radiation use efficiency of cotton (*Gossypium hirsutum* L.) under repeated transient waterlogging in the field. Field Crops Res. 2013; 140: 51–58.

[39] Ashraf MA, Ahmad MSA, Asharf M, Al-Qurainy F, Ashraf MY. Alleviation of waterlogging stress in upland cotton (*Gossypium hirsutum* L.) by exogenous application of potassium in soil and as a foliar spray. Crop Pasture Sci. 2011; 62: 25–38.

[40] Cao G, Wang X, Liu Y, Luo W. Effect of waterlogging stress on cotton leaf area index and yield. Procedia Eng. 2012; 28(12): 202–209.

[41] Bange MP, Milroy SP. Growth and dry matter partitioning of diverse cotton genotypes. Field Crops Res. 2004; 87: 73–87.

[42] Bange MP, Milroy SP, Thongbai P. Growth and yield of cotton in response to waterlogging. Field Crops Res. 2004; 88: 129–142.

[43] Najeeb U, Bange MP, Daniel KYT, Atwell BJ. Consequences of waterlogging in cotton and opportunities for mitigation of yield losses. Valuation of corporate growth opportunities: Garland Pub. 2015; 55–68. 10.1093/aobpla/plv080 PMC4565423 26194168

[44] Hodgson AS. The effects of duration: timing and chemical amelioration of short-term waterlogging during furrow irrigation of cotton in a cracking grey clay. Aust. J. Agric. Res. 1982; 33: 1019–1028.

[45] Kuai J, Zhou Z, Wang Y, Meng Y, Chen B, Zhao W. The effects of short-term waterlogging on the lint yield and yield components of cotton with respect to boll position. Eur J Agron. 2015; 67: 61–74.

[46] Luan J, Li J, Varela N, Wang Y, Li F, Bao Y, et al Global analysis of the transcriptional response of whitefly to tomato yellow leaf curl china virus reveals the relationship of coevolved adaptations. J Virol. 2011; 85: 3330–3340. doi: 10.1128/JVI.02507-10 21270146 10.1128/JVI.02507-10 PMC3067855

[47] Li H, Ruan J, Durbin R. Mapping short DNA sequencing reads and calling variants using mapping quality scores. Genome Res.2008; 18: 1851–1858. doi: 10.1101/gr.078212.108 18714091 10.1101/gr.078212.108 PMC2577856

[48] Wu T, Qin ZW, Zhou XY, Feng Z, Du YL. Transcriptome profile analysis of floral sex determination in cucumber. J Plant Physiol. 2010; 167: 905–913. doi: 10.1016/j.jplph.2010.02.004 20303197 10.1016/j.jplph.2010.02.004

[49] Mortazavi A, Williams BA, McCue K, Schaeffer L, Wold B. Mapping and quantifying mammalian transcriptomes by RNA-Seq. Nat Methods. 2008; 5: 621–628. doi: 10.1038/nmeth.1226 18516045 10.1038/nmeth.1226 PMC13303166

[50] Audic S, Claverie JM. The significance of digital gene expression profiles. Genome Res. 1997; 7: 986–995. 9331369 10.1101/gr.7.10.986

[51] Du Z, Zhou X, Ling Y, Zhang Z, Su Z. AgriGO: a GO analysis toolkit for the agricultural community. Nucleic Acids Res. 2010; 38: 64–70. 10.1093/nar/gkq310 PMC2896167 20435677

[52] Tang QY, Feng MG. Practical statistics and DPS data processing system In: Tang QY, Feng MG, editors. DPS Data Processing System for Practical Statistics. China Agricultural Press; 1997 pp. 188–195.

[53] Zhang Y, Song X, Yang G, Li Z, Lu H, Kong X, et al Physiological and molecular adjustment of cotton to waterlogging at peak-flowering in relation to growth and yield. Field Crops Research. 2015; 179: 164–172.

[54] Zheng CF, Jiang D, Liu FL. Effects of salt and waterlogging stresses and their combination on leaf photosynthesis, chloroplast ATP synthesis, and antioxidant capacity in wheat. Plant Science. 2009; 176(4): 575–582. doi: 10.1016/j.plantsci.2009.01.015 26493148 10.1016/j.plantsci.2009.01.015

[55] Lee YH, Kim KS, Jang YS. Global gene expression responses to waterlogging in leaves of rape seedlings. Plant Cell Reports. 2014; 33(2): 289–99. doi: 10.1007/s00299-013-1529-8 24384821 10.1007/s00299-013-1529-8

[56] Luo Z, Dong HZ, Li WJ, Zhao M, Zhu YQ. Individual and combined effectsof salinity and waterlogging on Cry1Ac expression and insecticidal efficacy of Bt cotton. Crop Prot. 2008; 27: 1485–1490

[57] Geigenberger P, Fernie AR, Gibon Y, Christ M, Stitt M. Metabolic activity decreases as an adaptive response to low internal oxygen in growing potato tubers, Biol. Chem. 2000; 381: 723–740. doi: 10.1515/BC.2000.093 11030430 10.1515/BC.2000.093

[58] Bologa KL, Fernie AR, Leisse A, Loureiro ME, Geigenberger P. A bypass of sucrose synthase leads to low internal oxygen and impaired metabolic performance in growing potato tubers, Plant Physiol. 2003; 132: 2058–2072. doi: 10.1104/pp.103.022236 12913161 10.1104/pp.103.022236 PMC181290

[59] Rivoal J, Thind S, Pradet A, Ricard B. Differential induction of pyruvate decarboxylase subunits and transcripts in anoxic rice seedlings. Plant Physiology. 1997; 114(3): 1021–9. 9232881 10.1104/pp.114.3.1021 PMC158390

[60] Mithran M, Paparelli E, Novi G, Perata P, Loreti E. Analysis of the role of the pyruvate decarboxylase gene family in Arabidopsis thaliana under low-oxygen conditions. Plant Biology. 2014; 16(1): 28–34. doi: 10.1111/plb.12005 23574450 10.1111/plb.12005

[61] Sairam RK, Kumutha D, Chinnusamy V, Meena RC. Waterlogging-induced increase in sugar mobilization, fermentation and related gene expression in roots of mungbean (*Vigna radiata*). J Plant Physiol. 2009; 166: 602–616 doi: 10.1016/j.jplph.2008.09.005 18947901 10.1016/j.jplph.2008.09.005

[62] Kumutha D, Sairam RK, Ezhilmathi K, Chinnusamy V, Meena RC. Effect of waterlogging on carbohydrate metabolism in pigeon pea (*Cajanus cajan* L.): Upregulation of sucrose synthase and alcohol dehydrogenase. Plant Sci. 2008; 175: 706–716

[63] Gibbs J, Morrell S, Valdez A, Selter TL, Greenway H. Regulation of alcoholic fermentation in coleoptiles of two rice cultivars differing in tolerance to anoxia. J Exp Bot. 2000; 51: 785–796 10938871

[64] Drew MC. Oxygen deficiency and root metabolism. Injury and acclimation under hypoxia and anoxia. Annu Rev Plant Physiol Mol Biol. 1997; 48: 223–250 10.1146/annurev.arplant.48.1.223 15012263

[65] Muench DG, Archibold OW, Good AG. Hypoxic metabolism in wild rice (*Zizania palustris*): Enzyme induction and metabolic production. Physiol Plant. 1993; 89: 165–171

[66] Kato-Noguchi H. Evaluation of the importance of lactate for the activation of ethanolic fermentation in the lettuce in anoxia. Physiol Plant. 2000; 109: 28–33

[67] Setter TL, Ella ES, Valdez AP. Relationship between coleoptile elongation and alcoholic fermentation in rice exposed to anoxia. II Cultivar differences. Ann Bot. 1994; 74: 273–279

[68] Perata P, Guglieminetti L, Alpi A. Mobilization of endosperm reserves in cereal seeds under anoxia. Ann Bot. 1997; 79: 49–56

[69] Jain V, Singla NK, Jain S. Activities of enzymes of fermentation pathways in the leaves and roots of contrasting cultivars of sorghum (*Sorghum Bicolor* L.) during flooding. Physiology and Molecular Biology of Plants. 2010; 16(3): 241–247. doi: 10.1007/s12298-010-0025-7 23572974 10.1007/s12298-010-0025-7 PMC3550673

[70] Roberts JKM, Jardetzky O. Mechanism of Cytoplasmic pH Regulation in Hypoxic Maize Root Tips and Its Role in Survival under Hypoxia. Proceedings of the National Academy of Sciences. 1984; 81(11): 3379–83. 10.1073/pnas.81.11.3379 PMC345511 6587355

[71] Ellis MH, Millar AA, Llewellyn DJ, Peacock WJ, Dennis ES. Transgenic cotton (*Gossypium hirsutum*) over-expressing alcohol dehydrogenase shows increased ethanol fermentation but no increase in tolerance to oxygen deficiency. Functional Plant Biology. 2000; 27(11): 1041–1050.

[72] Hamonts K, Clough TJ, Stewart A, Clinton PW, Richardson AE, Wakelin SA, et al Effect of nitrogen and waterlogging on denitrifier gene abundance, community structure and activity in the rhizosphere of wheat. FEMS Microbiol Ecol. 2012; 83(3): 568–84. doi: 10.1111/1574-6941.12015 23006139 10.1111/1574-6941.12015

[73] Shi HT, Li RJ, Cai W, Liu W, Wang CL, Lu YT. Increasing nitric oxide content in Arabidopsis thaliana by expressing rat neuronal nitric oxide synthase resulted in enhanced stress tolerance. Plant and Cell Physiology. 2012; 53(2): 344–57. doi: 10.1093/pcp/pcr181 22186181 10.1093/pcp/pcr181

[74] Kaiser WM. On the origins of nitric oxide. Trends in Plant Science. 2010; 16(3): 160–8. doi: 10.1016/j.tplants.2010.11.007 21185769 10.1016/j.tplants.2010.11.007

[75] Gill SS, Hasanuzzaman M, Nahar K, Macovei A, Tuteja N. Importance of nitric oxide in cadmium stress tolerance in crop plants. Plant Physiology & Biochemistry. 2012; 63C (4): 254–261. 10.1016/j.plaphy.2012.12.001 23313792

[76] Ding F, Wang XF, Shi QH, Wang ML, Yang FJ, Gao QH. Exogenousnitric oxide alleviated the inhibition of photosynthesis and antioxidant enzyme activities in iron-deficient Chinese cabbage (*Brassica chinensis* L.). Agric Sci. China. 2008; 7: 168–179.

[77] Horchani F, Aschi-Smiti S, Brouquisse R. Involvement of nitrate reduction in the tolerance of tomato (*Solanum lycopersicum* L.) plants to prolonged root hypoxia. Acta Physiol. Plant. 2010; 32: 1113–1123.

[78] Oliveira HC, Freschi L, Sodek L. Nitrogen metabolism and translocation in soybean plants subjected to root oxygen deficiency. Plant Physiol. Biochem. 2013; 66: 141–149. doi: 10.1016/j.plaphy.2013.02.015 23500717 10.1016/j.plaphy.2013.02.015

[79] Oliveira HC, Sodek L. Effect of oxygen deficiency on nitrogen assimilation and amino acid metabolism of soybean root segments. Amino Acids. 2013; 44:743–755. doi: 10.1007/s00726-012-1399-3 22990842 10.1007/s00726-012-1399-3

[80] Chen T, Yuan F, Song J, Wang B. Nitric oxide participates in waterlogging tolerance through enhanced adventitious root formation in the euhalophyte *Suaeda salsa*. Functional Plant Biology. 2016; 43(3). 10.1071/FP15120 32480457

[81] Song XZ. Effects of SNP on relieving cotton damage caused by waterlogging during boll setting period. M.Sc. Thesis, Huazhong Agriculture University.2013.

[82] Allegre A, Silvestre J, Morard P, Kallerhoff J, Pinelli E. Nitrate reductase regulation in tomato roots by exogenous nitrate: a possible role in tolerance to long-term root anoxia. J. Exp. Bot. 2004; 55: 2625–2634. doi: 10.1093/jxb/erh258 15475378 10.1093/jxb/erh258

[83] Stohr C, Strube F, Marx G, Ullrich WR, Rockel P. A plasma membrane-bound enzyme of tobacco roots catalyses the formation of nitric oxide from nitrite. Planta. 2001; 212(5–6): 835–841. 11346959 10.1007/s004250000447

[84] Musgrave A, Jackson MB, Long E. Gallitniche stem elongation is controlled by ethylene and gibberellin. Nature New Biology. 1972; 238: 93–96.

[85] Kende H, van der Knaap E, Cho HT. Deepwater rice: a model plant to study stem elongation. Plant Physiol. 1998; 118: 1105–1110 9847084 10.1104/pp.118.4.1105 PMC1539197

[86] Voesenek LA, Benschop JJ, Bou J, Cox MCH, Greoneveld HW, Millenaar FF, et al Interactions between plant hormones regulate submergence-induced shoot elongation in the flooding-tolerant dicot Rumex palustris. Ann Bot. 2003; 91: 205–211. doi: 10.1093/aob/mcf116 12509341 10.1093/aob/mcf116 PMC4244986

[87] Voesenek LACJ, Rijnders JHGM, Peeters AJM, Van de Steeg HMV, De Kroon H. Plant hormones regulate fast shoot elongation under water: from genes to communities. Ecology. 2004; 85: 16–27

[88] Pierik R, Millenaar FF, Peeters AJM. New Perspectives in Flooding Research: the Use of Shade Avoidance and Arabidopsis thaliana. Annals of Botany. 2005; 96(4): 533–40. doi: 10.1093/aob/mci208 16027134 10.1093/aob/mci208 PMC4247023

[89] Benschop JJ, Bou J, Peeters AJM. Long-term submergence-induced elongation in Rumex palustris requires abscisic acid-dependent biosynthesis of gibberellin1. Plant Physiology. 2006; 141(4): 1644–52. doi: 10.1104/pp.106.082636 16766669 10.1104/pp.106.082636 PMC1533959

[90] Hoffmann-Benning S, Kende H. On the role of abscisic-acid and gibberellin in the regulation of growth in rice. Plant Physiol. 1992; 99: 1156–1161 16668983 10.1104/pp.99.3.1156 PMC1080597

[91] Fukao T, Yeung E, Bailey-Serres J. The submergence tolerance regulator SUB1A mediates crosstalk between submergence and drought tolerance in rice. Plant Cell. 2011; 23: 412–427. doi: 10.1105/tpc.110.080325 21239643 10.1105/tpc.110.080325 PMC3051255

[92] Gallego-Giraldo C, Hu J, Urbez C, Gomez MD, Sun T, Perez-Amador MA. Role of the gibberellin receptors gid1 during fruit-set in Arabidopsis. Plant Journal. 2014; 79(6): 1020–1032. doi: 10.1111/tpj.12603 24961590 10.1111/tpj.12603 PMC4403254

[93] Morris DA. The role of auxin in the apical regulation of leaf abscission in cotton (*Gossypium hirsutum* L.). J Exp Bot. 1993; 44(261): 807–814.

[94] Nguyen D, Rieu I, Mariani C, Van Dam NM. How plants handle multiple stresses: hormonal interactions underlying responses to abiotic stress and insect herbivory. Plant Molecular Biology. 2016; 91(6): 727–740. doi: 10.1007/s11103-016-0481-8 27095445 10.1007/s11103-016-0481-8 PMC4932144

[95] Yang CY, Hsu FC, Li JP, Wang NN, Shih MC. The AP2/ERF transcription factor AtERF73/HRE1 modulates ethylene responses during hypoxia in Arabidopsis. Plant Physiology. 2011; 156: 202–212. doi: 10.1104/pp.111.172486 21398256 10.1104/pp.111.172486 PMC3091062

[96] Licausi F, van Dongen JT, Giuntoli B, Novi G, Santaniello A, Geigenberger P, et al HRE1 and HRE2, two hypoxia-inducible ethylene response factors, affect anaerobic responses in Arabidopsis thaliana. The Plant Journal. 2010; 62: 302–315. doi: 10.1111/j.1365-313X.2010.04149.x 20113439 10.1111/j.1365-313X.2010.04149.x

